# Barley *TAPETAL DEVELOPMENT* and *FUNCTION1* (*HvTDF1*) gene reveals conserved and unique roles in controlling anther tapetum development in dicot and monocot plants

**DOI:** 10.1111/nph.19161

**Published:** 2023-08-10

**Authors:** Miaoyuan Hua, Wenzhe Yin, José Fernández Gómez, Alison Tidy, Guangwei Xing, Jie Zong, Shuya Shi, Zoe A. Wilson

**Affiliations:** ^1^ Division of Plant and Crop Sciences, School of Biosciences University of Nottingham Sutton Bonington Campus Loughborough Leics LE12 5RD UK; ^2^ School of Life Sciences and Biotechnology Shanghai Jiao Tong University Shanghai 200240 China; ^3^ Empyrean Neuroscience Cambridge CB22 3AT UK; ^4^ Goethe University Frankfurt am Main Max‐von‐Laue Str. 9 Frankfurt am Main 60438 Germany

**Keywords:** anther, barley, fertility, male sterility, osmotin, tapetum, TDF1, transcriptome

## Abstract

- The anther tapetum helps control microspore release and essential components for pollen wall formation. TAPETAL DEVELOPMENT and FUNCTION1 (TDF1) is an essential R2R3 MYB tapetum transcription factor in *Arabidopsis thaliana*; however, little is known about pollen development in the temperate monocot barley.
- Here, we characterize the barley (*Hordeum vulgare* L.) *TDF1* ortholog using reverse genetics and transcriptomics.
- Spatial/temporal expression analysis indicates *HvTDF1* has tapetum‐specific expression during anther stage 7/8. Homozygous barley *hvtdf1* mutants exhibit male sterility with retarded tapetum development, delayed tapetum endomitosis and cell wall degeneration, resulting in enlarged, vacuolated tapetum surrounding collapsing microspores. Transient protein expression and dual‐luciferase assays show TDF1 is a nuclear‐localized, transcription activator, that directly activates osmotin proteins. Comparison of *hvtdf1* transcriptome data revealed several pathways were delayed, endorsing the observed retarded anther morphology. Arabidopsis *tdf1* mutant fertility was recovered by *HvTDF1*, supporting a conserved role for *TDF1* in monocots and dicots.
- This indicates that tapetum development shares similarity between monocot and dicots; however, barley *HvTDF1* appears to uniquely act as a modifier to activate tapetum gene expression pathways, which are subsequently also induced by other factors. Therefore, the absence of *HvTDF1* results in delayed developmental progression rather than pathway failure, although inevitably still results in pollen degeneration.

## Introduction

Viable pollen is critical for successful fertilization in the plant. The tapetum is the innermost layer of the anther wall, which directly communicates with developing pollen grains, and finally degenerates at the mature pollen grain stage. It is involved in the production of the enzymes for releasing microspores from the tetrads and providing essential nutrients for pollen wall and coat formation (Goldberg *et al*., 1993). Several key transcription factors regulate tapetum development and have been studied in model plants, such as Arabidopsis (Wilson *et al*., 2001; Ito & Shinozaki, 2002; Sorensen *et al*., 2003; Zhang *et al*., 2006, 2007; Zhu *et al*., 2008; Phan *et al*., 2011); some of their orthologous genes have also reported in crop plants, such as rice, barley and maize (Fernández Gómez *et al*., 2015). Based on studies of mutant lines, the regulatory relationship between them has been preliminarily established and suggests a relatively conserved genetic pathway, *DYT1‐TDF1‐AMS‐MYB80/MS188‐MS1*, controlling tapetum development from early to late anther stages (Higginson *et al*., 2003; Zhang *et al*., 2006, 2007; Yang *et al*., 2007; Feng & Dickinson, 2010; Xu *et al*., 2010; Phan *et al*., 2011; Feng *et al*., 2012; Ferguson *et al*., 2017).

The characterization of molecular gene networks in crops is recognized as extremely challenging and consequently has been slow to provide the insight seen in model systems; however, whole genome sequencing and the updated transcriptome assembly in barley are now providing valuable tools for gene identification. This will expand opportunities to generate male sterile lines for hybrid breeding that correspond to the orthologous genes in barley. For example, the tapetum *MALE STERILITY 1* (*MS1*) gene, which encodes for a plant homeodomain (PHD) finger motif transcription factor (Wilson *et al*., 2001; Ito *et al*., 2007), was reported in Arabidopsis, and its rice orthologous gene *OsPTC1* was subsequently characterized (Li *et al*., 2011). Based on the conserved role of *MS1* genes in Arabidopsis and rice, Fernández Gómez and Wilson (2014) using a reverse genetic approach, reported the first barley male sterility gene, *HvMS1*.

The *TDF1* gene encodes for a R2R3 MYB tapetum transcription factor, which was studied first in Arabidopsis (Zhu *et al*., 2008). Further work showed it directly regulates the expression of the master tapetum regulator, *ABORTED MICROSPORES* (*AMS*; Lou *et al*., 2018). TDF1 forms a complex with AMS to regulate gene expression through a feed‐forward loop, which includes activating the essential tapetum and pollen grain development gene *EPXB5* (Lou *et al*., 2018). Additionally, the study of *OsTDF1* gene in rice further indicates a potential conserved role in monocot and dicot plants (Cai *et al*., 2015). Nevertheless, studies have indicated that some potentially conserved genes are showing distinct functions in species separated by long evolutionary distances, as there are copy number differences and/or divergent functions observed. For example, the wheat *TaMS1* gene was characterized as a key factor in wheat pollen wall formation, and its orthologous gene, *EPAD1*, was also reported in rice. Both appear to show the same function in pollen wall patterning (Tucker *et al*., 2017; Wang *et al*., 2017; Li *et al*., 2021); however, there are three copies of *TaMS1* in wheat and one in rice. The latest study of the two *TaMS1* orthologues gene in maize showed that the maize genes cannot rescue fertility in wheat, suggesting functional divergence (Li *et al*., 2021). It is also becoming increasingly evident that tapetum transcriptional control is not a direct pathway; rather, it involves multiple feedback loops which may be regulated differently between species (Ferguson *et al*., 2017).

Here, we have identified and characterized the barley *HvTDF1* gene via reverse genetic approaches. *HvTDF1* recovers fertility in the Arabidopsis *tdf1* mutant, suggesting that *TDF1* genes may play a conserved role in dicots and monocots. HvTDF1 protein was shown to activate gene expression by a modified dual‐luciferase reporter (DLR) assay. CRISPR‐Cas9 generated barley *hvtdf1* mutants were male sterile due to pollen degeneration. Semi‐thin sections of *hvtdf1* anthers showed initial tapetum cell fate determination, including tapetum cell wall degeneration and tapetum endomitosis; however, the mutant required longer to complete tapetum cell fate determination. During the subsequent development stages, the mutant tapetum was enlarged with vacuolated cells containing some irregular round vacuole‐like structures. Callose degeneration also appeared abnormal and persisted during the defective tapetum development process in *hvtdf1*. RNA‐seq analysis of anther development indicated that several pathways showed delayed expression in *hvtdf1*. Gene Ontology (GO) term analysis on the down‐regulated genes from *hvtdf1* and *attdf1*, respectively, indicated that TDF1‐related downstream pathways are highly conserved among barley and Arabidopsis; however, in the *hvtdf1* mutant, the expression of these pathways was delayed not absent. In summary, our data suggest that the TDF1 tapetum regulatory genetic pathway may be conserved in dicots and monocots and act as a key determinant of tapetum differentiation and development essential for viable barley pollen formation. However, our detailed comparative analysis indicates a nonlinear regulation pathway for pollen development suggesting that other factors also regulate these gene networks leading to the complex control of gene expression needed for functional pollen development. Such knowledge provides essential detail to enable comparisons between species, which is necessary to enable the translation of knowledge from studies in model plants, such as Arabidopsis, to crops.

## Materials and Methods

### Plant materials and growth conditions

Two‐row spring barley (*Hordeum vulgare* L.) cultivar, Golden Promise (Fernández Gómez & Wilson, 2012), was used as the wild‐type (WT) control and for transformation to generate CRISPR‐Cas9 knock‐out lines. Transgenic and WT plants were grown under 15°C/12°C; 16‐h photoperiod; 80% RH, 500 μmol m^−2^ s^−1^ metal halide lamps (HQI) supplemented with tungsten bulbs. Seeds were sown in 12‐well pots (John Innes No. 3 compost) and after 2–3 wk transferred into 5‐l pots (Levington CNSC compost; three plants/pot).

### Molecular cloning of *HvTDF1* and polygenetic tree analysis

Total cDNA was prepared from different spike stages from Golden Promise and used to amplify *HvTDF1* coding sequence (Supporting Information Table S1 for primers) with Phusion high‐fidelity polymerase (F630L; ThermoFisher Scientific, Heysham, UK). HvTDF1 protein sequence was used to perform polygenetic tree analysis with other orthologous genes. Protein alignment was conducted by ClustalW, and polygenetic tree was constructed by neighbour‐joining.

### *HvTDF1* complementation assay in the Arabidopsis *tdf1* mutant background

To generate the TDF1 complementation assay vector, pBGWSF7 expression vector was linearized (KpnI‐HF and SpeI‐HF). The *Arabidopsis thaliana* (L.) Heynh., *TDF1* promoter (1.23 kb), was amplified and inserted into linearized pBGWSF7 by CloneExpress II One Step Cloning (C112; Vazyme, https://www.vazymebiotech.com) to generate pBGWSF7:AtTDF1pro:GW vector. Primers are detailed in Table S1. The ligated vector was transformed into ccdb survival *Escherichia coli* strain (Invitrogen). The *HvTDF1* CDS was inserted into the vector via a LR reaction. The generated expression vector, pBGWSF7:AtTDF1pro:HvTDF1cds, was transformed into *Agrobacterium* GV3101 and transformed into Arabidopsis *tdf1*± heterozygous mutant background by floral dipping (Fernández Gómez & Wilson, 2014). Seeds from the T0 generation were sown and screened with BASTA. Putative HvTDF1 transgenic plants were confirmed by PCR and Sanger sequencing to identify the *attdf1* homozygous background. Pollen viability was determined by Alexander staining according to Fernández Gómez and Wilson (2014).

### Generating barley *HvTDF1* CRISPR Cas9 knock‐out mutants

*HvTDF1* CRISPR‐Cas9 sgRNA targets were selected using www.deskgen.com and screened against the barley genome database to minimise off‐targets. CRISPR‐Cas9 vector generation and barley transformation were based upon Bartlett *et al*. (2008) and Lawrenson *et al*. (2015), respectively. The target region was amplified with 6880F/6882R (Table S1) using Red‐Taq (733‐2131; VWR, Lutterworth, UK) and Sanger sequencing to identify mutations.

### Protein transient expression and subcellular localization analysis of *HvTDF1*

*HvTDF1* coding sequence without stop‐codon was amplified and inserted into pCR™8/GW/TOPO™ vector to generate a pENTRY vector (Table S1). HvTDF1 coding region with the flanking attL1 and the attL2 region was amplified by Phusion® High‐Fidelity DNA Polymerase (Primers 1857, 1858; Table S1) using pCR8GW: HvTDF1‐nonstop ENTRY vector as template. The purified PCR product was used to generate pUbi10pro:HvTDF1‐GFP, the sequence‐confirmed vector was co‐transformed with pBIN‐P19 into *Agrobacterium* GV3101. A positive *Agrobacterium* colony was cultured overnight to OD_600_ 0.4–1 with Spectinomycin (100 mg ml^−1^), Kanamycin (50 mg ml^−1^) and Rifampicin (50 mg ml^−1^) in 1 : 1000 dilution, pelleted and resuspended in infiltration buffer and infiltrated into 4‐ to 6‐wk‐old *Nicotiana benthamiana* Domin, leaves (Cui *et al*., 2016). After 48 h, infiltrated leaves were analysed for fluorescence signal expression (Leica DM4B; Leica Microsystems, Milton Keynes, UK); confirmation of protein subcellular localization was conducted by infiltration with 4,6‐diamidino‐2‐phenylindole (DAPI; 10236276001; Sigma‐Aldrich; 10–20 mins; 10 μg ml^−1^). DAPI and green fluorescent protein (GFP) were observed using a Leica DM4B microscope (DAPI: filter cube LED365; GFP: filter cube LED470).

### Determining the expression pattern of *HvTDF1* transcript by RT‐qPCR and RNA *in situ* hybridization

Total mRNA was extracted from barley spikes at different anther stages (RNeasy; Qiagen); 3 μg total RNA was used to generate cDNA by Superscript™ IV VILO™ Master Mix with ezDNase™ Enzyme (Invitrogen™). RT‐qPCR was performed on a Roche LightCycler® 480 Instrument II platform (50°C for 2 min; 95°C for 2 min; 40 cycles 95°C for 15 s; 60°C for 15 s, 72°C for 1 min) using Applied Biosystems PowerUp SYBR Green Master Mix (ThermoFisher Scientific), with at least two biological replicates. Barley *α‐tubulin* and *Hsp70* were used as internal reference genes to normalize target gene expression levels. RNA *in situ* hybridization was according to Fernández Gómez and Wilson (2014).

### Modified dual‐luciferase reporter transcription factor activation assay

The *TDF1* coding region was fused with the GAL4‐DB domain, under regulation of the AtUBi10 promoter, to generate AtUBi10pro‐GAL4‐DB‐HvTDF1 expression cassette. In the pGreen800‐MCS‐LUC reporter vector, the Gal4‐UAS sequence was inserted into the Multiple Cloning Site (MCS), facilitating GAL4‐DB‐TF binding. These two vectors were transformed into *Agrobacterium* GV3101. pUB‐GAL4‐DB‐TF was co‐transformed with pBIN‐P19, while the pGreen800‐GAL4‐UAS‐LUC was co‐transformed with pSoup. Liquid cultures of the two vectors were mixed and infiltrated into 4‐ to 6‐wk‐old *N*. *bethamiana* leaves. After 2 days, both Firefly and Renilla luciferases were extracted from the infiltrated region by Passive Lysis buffer (E1941; Promega, manufacturer's instructions) and analysed using the Dual‐Glo Luciferase Assay System (Promega) and Synergy LX Multi‐Mode Reader.

To look for direct activation of potential TDF1 targets, the following constructs were created, AtUBi10pro‐HvTDF1‐GFP expression cassette, and in pGreen0802‐mini35S‐LUC reporter vector two putative binding sites per gene (AMS, OSMOTIN 1/2/3) were inserted to create eight reporter vectors. The transformation and Luc assay was the same as above.

### Phenotypic analyses of the *hvtdf1* mutants

Plants and floral organs were photographed using a Panasonic DMC‐GX80 and Zeiss Stemi 508 (Carl Zeiss Ltd, Cambridge, UK), respectively. Floral samples for semi‐thin sectioning and TEM were prepared as described previously (Fernández Gómez *et al*., 2020). For Scanning Electron Microscopy (SEM), samples were processed by methanol–ethanol fixation, followed by critical point drying (Leica EM CPD300), coated with gold by sputter coating (nmRC; University of Nottingham) and then examined using a JEOL 6060LV SEM (Talbot & White, 2013). Fixed samples for callose staining were embedded in paraffin; 6 μm transverse sections was stained with 0.05% aniline blue solution and examined with a Lecia SP5 confocal microscope as previously detailed (Vizcay‐Barrena & Wilson, 2006; Fu *et al*., 2014). Barley spike samples were fixed with Carnoy solution (EtOH: Glacial acetic acid = 3 : 1), meiosis cells observed for meiotic progression using DAPI (Sigma) after squashing and UV observation.

### Transcriptome sequencing and data analysis

Spikes from anther development stages 6–8b were collected from WT plants and spikes from stage 8a2 to 8b were collected from *hvtdf1‐2* mutants. Total RNA isolation, mRNA library preparation and sequencing were conducted by a custom service (BGI; Hong Kong). Sequence data were mapped to the barley Golden Promise reference transcriptome and quantified by Galaxy quantification pipeline (Schreiber *et al*., 2020; Guo *et al*., 2021). Read numbers from each sample were used to generate the reads matrix and analysed by iDEP.95 and ShinyGO (Ge *et al*., 2018, 2020). Blast analysis between the Arabidopsis and Barley genome was performed using CLC Genomics Workbench (Qiagen) to identify orthologues and further GO analysis was performed using agriGO v.2.0 (Tian *et al*., 2017). The heatmap figures of PGSEA pathways were generated by TBtools (Chen *et al*., 2020).

## Results

### Identification and isolation of barley *HvTDF1* by reverse genetics approaches

*TDF1* genes were previously reported in both Arabidopsis and rice (Zhu *et al*., 2008; Cai *et al*., 2015), suggesting a possible conserved role in controlling anther tapetum development in monocots and eudicots; however, no previous analysis in temperate monocot crops such as barley had been conducted. To enable identification of the barley *TDF1* orthologous gene, we used bridging species to span the evolution distance across the grass species and help find the most likely *HvTDF1* candidate.

Pfeifer *et al*. (2013) conducted a study on the evolutionary distance among perennial ryegrass, barley, *Brachypodium*, rice and sorghum, and the results showed close divergence time, 22–32 Ma, between barley and *Brachypodium*. The genomes of barley and *Brachypodium* show similar synonymous substitution rates on coding genes compared with rice (Pfeifer *et al*., 2013), suggesting a much closer evolutionary relationship between barley and *Brachypodium*. Therefore, *Brachypodium* was used as a bridging species to identify HvTDF1. First, the OsTDF1 amino acid (aa) sequence was screened against the *Brachypodium* database (https://plants.ensembl.org/Brachypodium_distachyon/Info/Index). The aa sequence BRADI_1g65238v3 (BdTDF1) was identified and used to screen the barley database, HORVU.MOREX.r2.4HG0319540 shares 81.79% aa sequence similarity. We also used HORVU.MOREX.r2.4HG0319540 to conduct reciprocal Blast analyses against *Brachypodium*, rice and Arabidopsis aa sequence databases, and the results showed that this barley gene product has the highest similarity score with BdTDF1, AtTDF1 and OsTDF1. Therefore, *HORVU*.*MOREX*.*r2*.*4HG0319540* was proposed as *HvTDF1* and utilized for further functional analysis and validation.

**Fig. 1:**
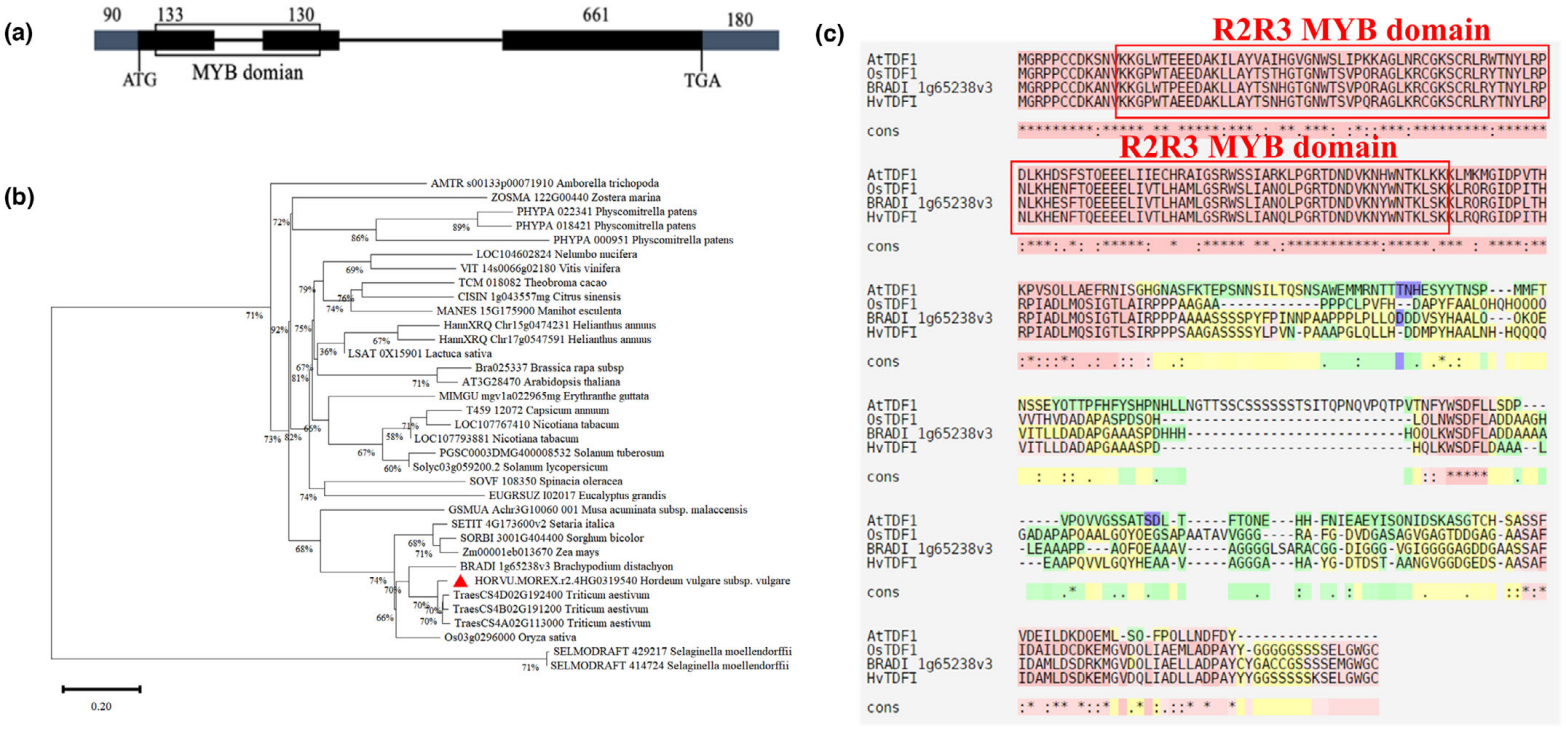
Gene structure of *HvTDF1* and phylogenetic analysis of *TDF1* genes from monocot and dicot species. (a) Schematic representation of *Hordeum vulgare TDF1* gene structure. Black box indicates exons, lines between exons indicate introns, grey boxes indicate 5′‐untranslated region (UTR) and 3′UTR, white box indicates the MYB domain. (b) Unrooted phylogenetic tree of HvTDF1 and orthologous genes using protein sequences from other mono‐ and dicot species, generated by Mega7 using the neighbour‐joining method. The gap data among all protein sequences were treated with partial deletion function and the amino acid site coverages (%) were listed at each node. The distance scale = 0.2. (c) Alignment of protein sequences of HvTDF1, *Arabidopsis thaliana* TDF1, *Brachypodium distachyon* TDF1 and *Oryza sativa* TDF1, generated by ClustalW, red box indicates the R2R3 MYB domain. Cons, conserved amino acids.

*AtTDF1/OsTDF1* encode for a R2R3 MYB superfamily transcription factor; the equivalent predicted conserved MYB domain was also seen in HvTDF1 N‐terminal region (Fig. 1a). HvTDF1 shares 47.46%, 81.10%, and 81.79% amino acid identity with AtTDF1, OsTDF1 and BdTDF1, respectively. Protein sequence alignment showed highest identity in the N‐terminal region of the R2R3 MYB domain (Fig. 1c). Phylogenetic tree constructed using the neighbour‐joining method on putative TDF1 aa sequences from diverse plant species showed clear diversification between the eudicot and monocot species; *HvTDF1* was classified in the same subclade with rice, wheat and *Brachypodium* within the monocot clade (Fig. 1b), confirming the close evolutionary relationship of *TDF1* among these species.

### Determining *HvTDF1* expression pattern

**Fig. 2:**
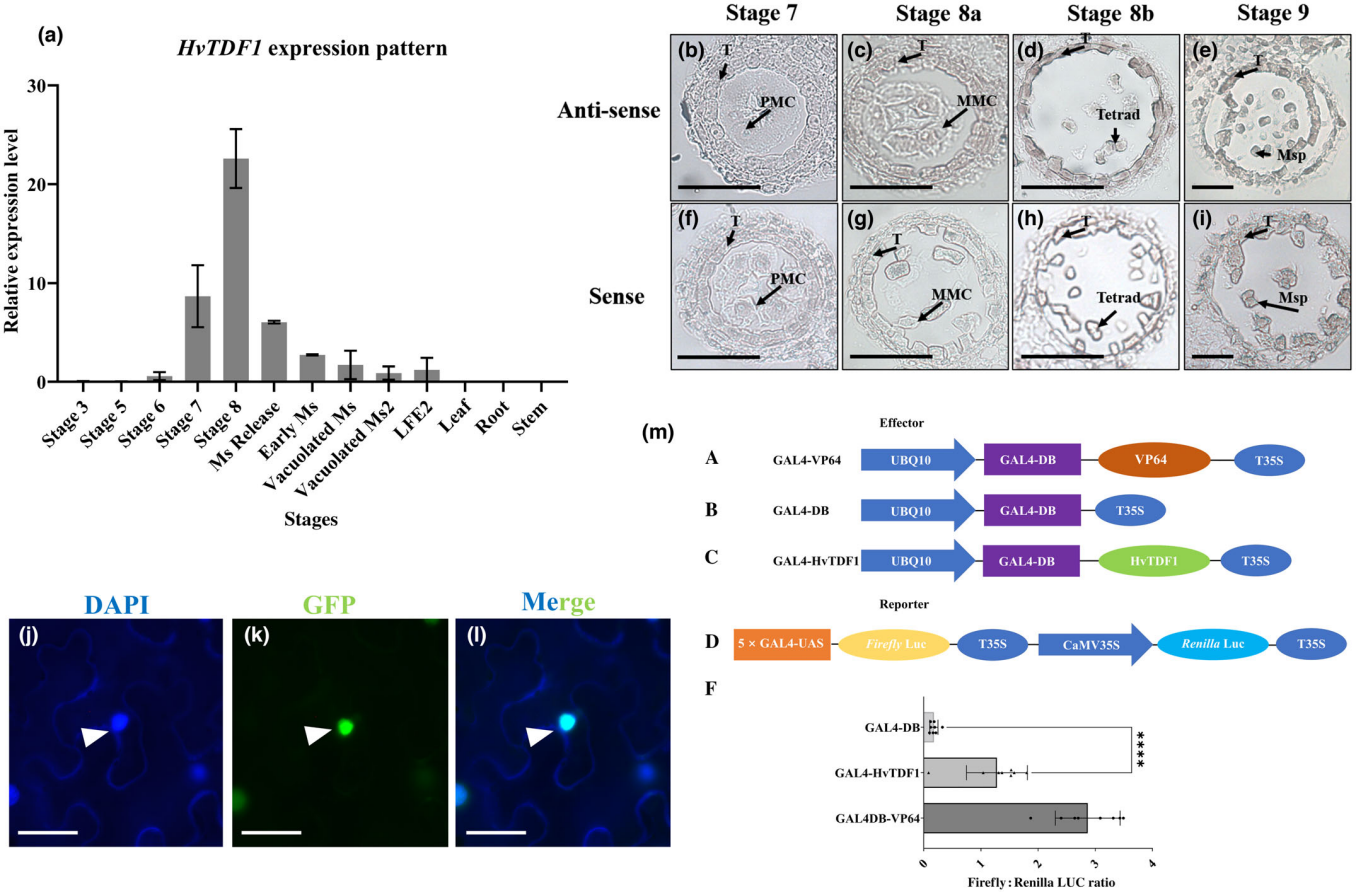
*HvTDF1* expression pattern and RNA *in situ* hybridization results of anther development stages in barley. (a) RT‐qPCR analysis of the relative expression of *Hordeum vulgare TDF1* from spikes at different anther development stages and other organs. The error bars indicating the standard errors from two replicates. (b–i) RNA *in situ* hybridization using a *HvTDF1*‐specific antisense probe (b–e) and a *HvTDF1* sense probe (f–i). (b, f) Secondary sporogenous cells to pollen mother cell stage. (c, g) Pollen mother cells undergoing meiosis, with the tapetum layer prominent. (d, h) Microspore release from the tetrad; tapetum vacuolated. (e, i) Free microspore stage; middle layer is crushed, the prominent tapetum layer starts to degenerate. T, tapetum; PMC, pollen mother cells; MMC, meiotic microspore cells; Msp, microspore. Bar, 50 μm. (j–l) Transient expressed GFP tagged HvTDF1 protein showing GFP signal (k) and DAPI stained nuclei (j) in *N*. *benthamiana* leaves showing the subcellular nuclear localization of HvTDF1 protein from merged channels (l) of DAPI and GFP (arrowhead). Bar, 25 μm. (m) Modified dual‐luciferase reporter (DLR) transcription factor activation assay on HvTDF1. The design of this yeast Gal4/UAS adopted DLR system (A–D). Features on vectors used to deliver the transient expression of effectors (A–C) and the dual‐reporters (D). The transactivation level of positive, HvTDF1 and negative effector presented as firefly : Renilla LUC ratio, which equals to the measured firefly luciferase luminescence normalised by the luminescence of the constitutively expressed Renilla luciferase (F). The GAL4‐DB‐VP64 (A) is an artificial activator as the positive control in this assay, showing strong activation ability compared the negative empty control, GAL4‐DB vector (F). The GAL4‐DB‐HvTDF1 fused protein showed significant higher expression ratio of Firefly and Renilla luciferase (F). VP64, a transcriptional activator composed of four tandem copies of VP16 (Herpes Simplex Viral Protein 16); Luc, luciferase; UBQ10, Arabidopsis Ubiquitin 10 promoter; 5xGal4UAS, synthetic sequence of five tandem copies of yeast Gal4 upstream activating sequence; T35S, Cauliflower Mosaic Virus (CaMV) 35S terminator. Error bars represent ± SE for eight biological replicates. Asterisk symbols, *t*‐test; *P* < 0.0001.

Arabidopsis and rice *TDF1* genes have similar expression patterns, which is limited to the anther tapetum during early tapetum development (Zhu *et al*., 2008, 2011; Cai *et al*., 2015). The expression pattern of *HvTDF1* in barley was determined by RT‐qPCR, and anthers were collected from the central zones of different spikes from different development stages based on the spike size (Gómez & Wilson, 2012) and additional tissues. *HvTDF1* starts to express in spikes from the Pollen Mother Cell (PMC) stage until the tetrad stage, and expression was rarely detected in later stages and not in the other organs assessed (Fig. 2a). RNA *in situ* hybridization was performed to identify spatial and temporal *HvTDF1* gene expression. Expression was detected in the tapetum from the Microspore Mother Cell (MMC) stage and tetrad (Fig. 2d,e), which corresponded to the RT‐qPCR results. *TDF1* genes encode for R2R3 MYB family transcription factors, which are predominantly nuclear localized in Arabidopsis and rice (Zhu *et al*., 2008; Cai *et al*., 2015). To establish protein localization of the barley TDF1, we performed transient protein expression in *N. benthamiana*. The *HvTDF1* coding region without stop codon was fused with GFP and used to detect subcellular localization; proUbi10:TDF1:GFP was only detected in nuclei (Fig. 2j–l) indicating conserved nuclear localization. The transcriptional activator role of the HvTDF1 protein was studied using a modified dual‐luciferase assay, which adopted the yeast Gal4/upstream activating sequence (UAS) system. The HvTFD1 was fused with yeast Gal4 DNA‐binding domain (Gal4‐DB) on an effector vector, co‐expressed in *N*. *benthamiana* leaves with a reporter vector possessing firefly luciferase driven by Gal4‐UAS. Alongside, a negative (sole Gal4‐DB) and a positive (Gal4‐DB fused with strong transcriptional activator VP64) effector control were co‐expressed, respectively, with the reporter vector, to define the baseline and saturated transactivation level. HvTDF1 was able to instigate positive regulation at a comparable (*c*. 50%) level to the Gal4‐DB‐VP64, which indicates HvTDF1 acts as an activator‐promoting expression of its regulatory target genes (Fig. 2m).

### Generating *HvTDF1* knock‐out mutant by CRISPR‐Cas9 gene editing

**Fig. 3:**
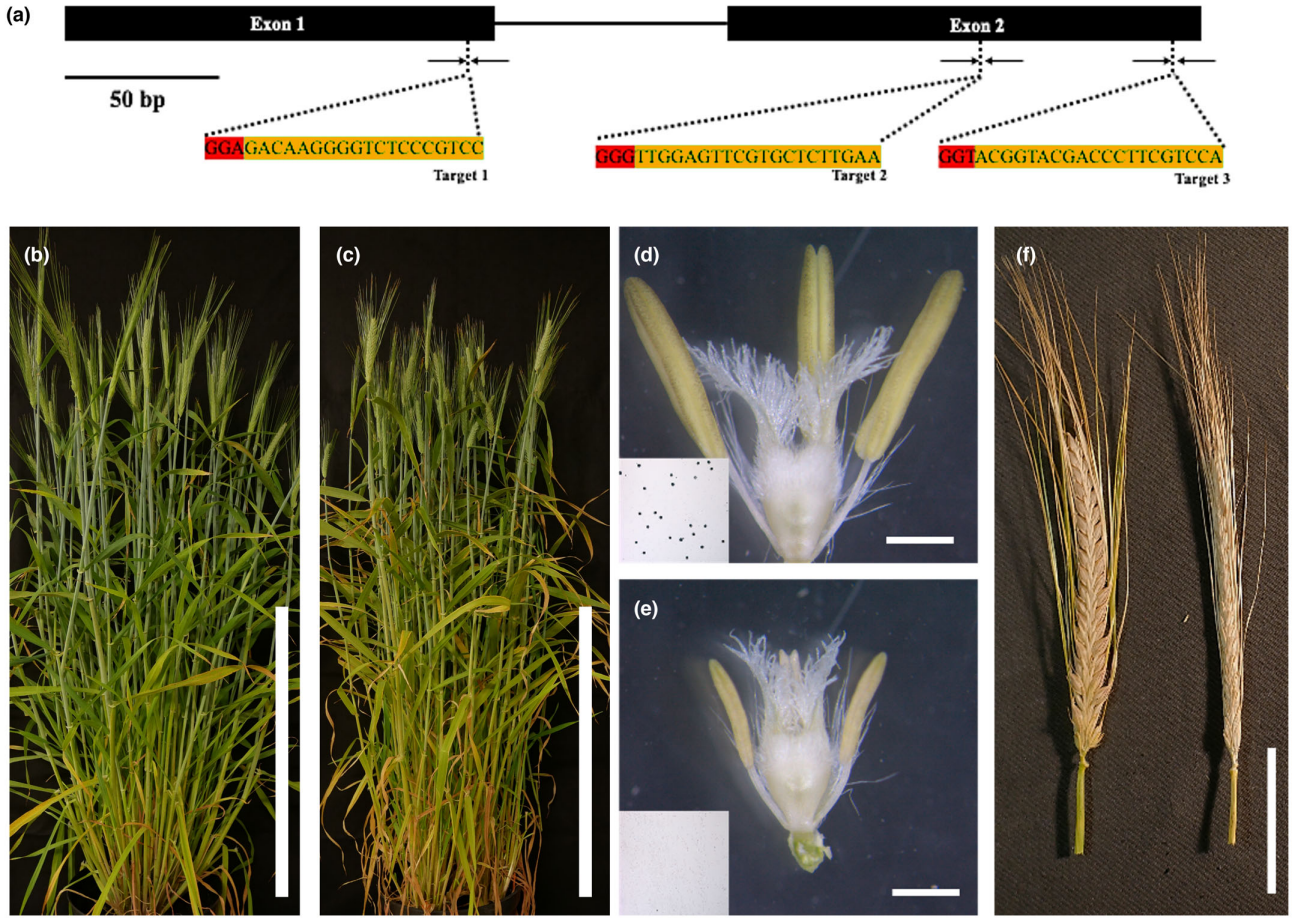
Characterization of *HvTDF1* CRISPR mutants. (a) Structure of *Hordeum vulgare TDF1* gene from exon 1 (130 bp) to exon 2 (131 bp), nuclei acid sequences underneath the gene structure are the sgRNA target regions (orange highlighting) and the protospacer‐adjacent motif (PAM) (red highlighting). (b) Wild‐type and (c) *hvtdf1‐2* plant morphology. (d) Wild‐type and (e) *hvtdf1‐2* floral tissues with removed lemma and palea, the inserted photos are KI–I_2_ stained pollen. (f) Wild‐type fertile (Left) and *hvtdf1‐2* (Right) sterile mature spikes. Bars: (a) 50 bp; (b, c) 50 cm; (d, e) 1 mm; (f) 5 cm.

Arabidopsis and rice *tdf1* knock‐out mutants resulted in male sterile anthers with vacuolated and enlarged tapetums (Zhu *et al*., 2008; Cai *et al*., 2015). A barley‐specific CRISPR/Cas9 system was used to generate knock‐out mutants of the *TDF1* barley gene. Three targets (Fig. 3a) were selected after screening against a barley database to minimise potential off‐target effects and assembled into a single multiple‐target CRISPR vector and transformed into *Agrobacterium* AGL1 (Lawrenson *et al*., 2015). In the T0 generation, 15 transgenic plants were generated, and the T‐DNA insertion confirmed by PCR (Fig. S1a). Seeds were collected from T0 plants and resown. Three independent alleles, *hvtdf1‐1*, *hvtdf1‐2* and *hvtdf1‐3*, were confirmed by Sanger sequencing, and these had different mutations including a single base insertion (*hvtdf1‐1*), a 237 bp deletion (*hvtdf1‐2*), and a single base insertion combined with a 56 bp deletion (*hvtdf1‐3*; Fig. S1b–e). To minimize the impact of any potential off‐target effects and confirm the specificity of the observed phenotypes, the homozygous mutants were backcrossed with Golden Promise, and maintained by self‐pollination until the T3 generation. All alleles showed the same general phenotypes (Figs 3c,e,f, S2b,c,e–g), homozygous *hvtdf1‐2* was analysed in detail and the deleted region further confirmed by PCR (Fig. S2h). General phenotyping showed that the barley homozygous mutant plants had normal vegetative growth and spike size, but no seed filling in mutant spikes (Fig. 3b,c,f). *hvtdf1‐2* plants had smaller anthers compared to WT, which lacked pollen grains (Fig. 3d,e). Genotyping indicated that segregation between WT and mutant plants was *c*. 3 : 1, suggesting that the phenotype was due to single recessive mutations.

### Barley *hvtdf1* mutants show defective tapetum development and degeneration

To characterize the anther locule defects, semi‐thin transverse sections of mutant anthers were analysed from the key development stages. Mature spike morphology in WT and *hvtdf1‐2* did not identify any differences in spike size. Previous studies showed a relationship between spike length and anther development stage, which can be used to accurately stage floral development progression (Fernández Gómez & Wilson, 2012). Thus, the same size spikes were collected from WT and *hvtdf1‐2* representing equivalent development stages.

**Fig. 4:**
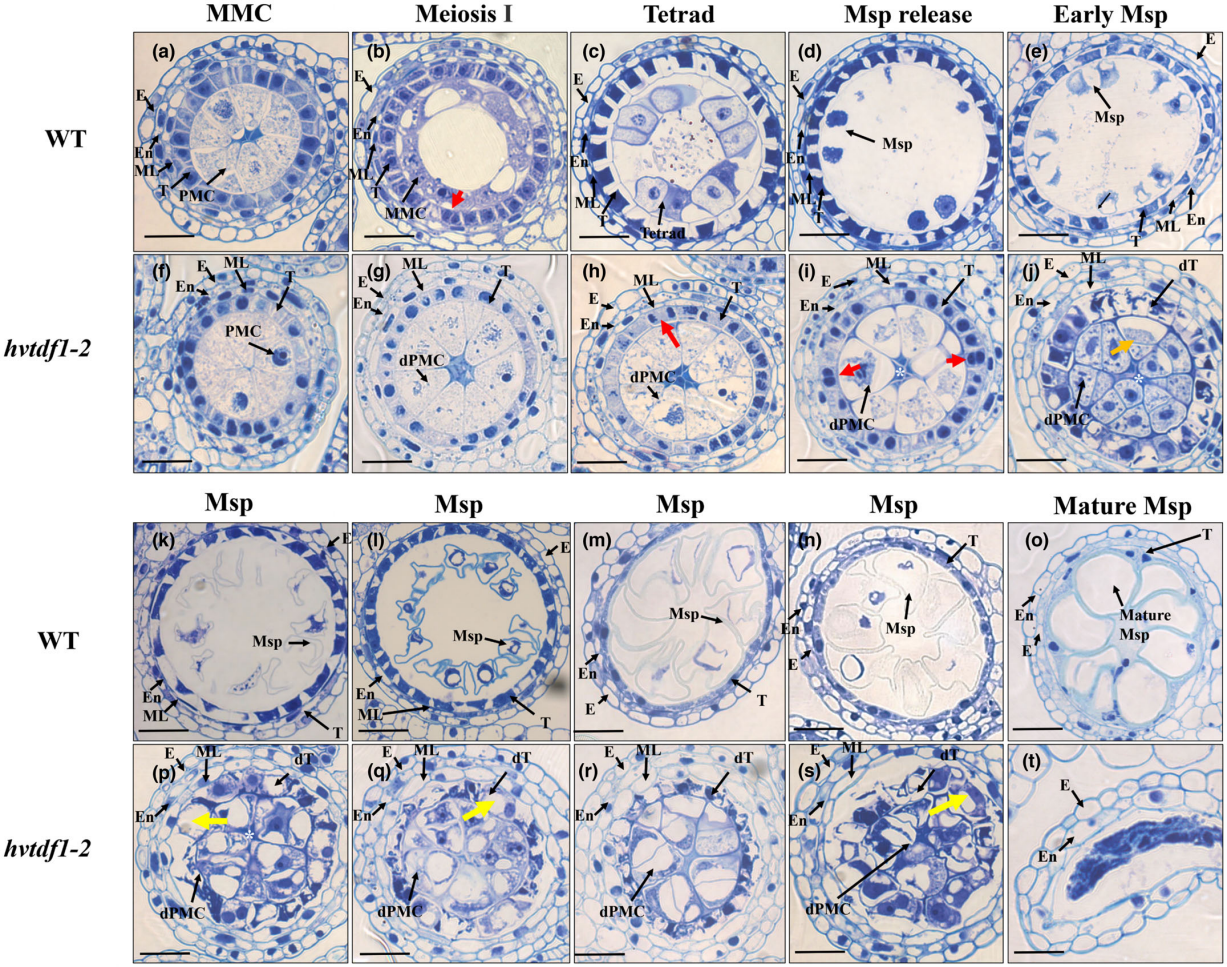
Transverse semi‐thin sections of wild‐type (WT) and the *hvtdf1‐2* mutant anthers from stage 7 to 9. (a–e, k–o) WT, (f–j, p–t) *Hordeum vulgare tdf1‐2*. (a, f) meiotic microspore cell stage 7, (b, g) meiosis I stage, (c, h) tetrad, (d, i) microspore release, (e, j) early microspore stage, (k–n, p–s) microspore stage, (o, t) mature microspore stage. dPMC, defective Pollen Mother Cell; dT, defective tapetum; E, epidermis; En, endothecium; ML, middle layer; MMC, Meiotic Microspore Cell; Msp, microspore; PMC, Pollen Mother Cell; T, tapetum. Red arrows indicate the endomitosis process within the tapetal cells, which occurs at meiosis I stage in WT but is delayed until tetrad‐microspore release stage in the *hvtdf1* mutant; Orange arrow (j) indicates irregular development of PMCs in *hvtdf1* which are halted at the dyad stage. Yellow arrows (p, q, s) indicate the vacuolated tapetum in *hvtdf1* mutant. Bar, 25 μm.

No obvious differences were observed between WT and mutant plant at stage 7, PMC stage (Fig. 4a,f). When the PMCs entered meiosis, the WT tapetum cells and meiotic microspore cells (MMCs) were deeply stained using 0.05% toluidine blue solution, indicating active biological processes (Fig. 4b). At this stage in WT, the tapetum cells start acquiring their specific cell fate, a key feature of this is endomitosis. However, at the same stage in *hvtdf1‐2*, both tapetum and defective pollen mother cells (dPMCs) showed faint toluidine blue staining, and the tapetum cells did not show detectable endomitosis and meiosis could not be observed in dPMCs (Fig. 4g). At tetrad stage, WT tapetum cells stained much darker and started the condensation process with tetrads attached to the inner tapetum surface (Fig. 4c). Meanwhile, delayed endomitosis was observed in the mutants, and spindles were visible in the tapetum cells (Fig. 4h); tapetum developmental progression appeared delayed and was similar to the meiosis stage in WT (Fig. 4b); however, the development of the dPMCs progressed at a similar pace as in WT PMC stage (Fig. 4a). The meiosis process of PMCs is not independent, it requires communication between germline cells and the surrounding tapetal cells, and they maintain equivalent developmental pace. We determined the chromosome behaviour of PMCs with DAPI staining during meiosis in WT and the *hvtdf1‐2* mutant. The results indicate that while the mutant PMCs had delayed meiosis compared with WT, meiosis I could successfully initiate in *hvtdf1‐2* (Fig. S3). At the microspore release stage in WT, the single microspore cells were released from the tetrads, and the tapetum layer was present and darkly stained (Fig. 4d). The mutant lines showed two re‐formed nuclei in the tapetum cells, indicating that endomitosis had occurred and that tapetum cells had acquired their specific cell fate (Fig. 4i). During microspore development stage in WT, the vacuoles in the microspores increased in size, resulting in round, mature microspores with off‐set nuclei, whilst the tapetum layer started degeneration and became thinner (Fig. 4e,k–o). However, in the mutant lines, the tapetum became vacuolated and stained less. The dyads formed in the mutant line at early single microspore stage became vacuolated during the subsequent development stages until the anther locule collapsed, and no obvious tetrads could be observed (Fig. 4j,p–t). The middle layer degenerated in WT (Fig. 4m), but remained as a vacuolated cell layer in the mutant (Fig. 4r,s). In previous Arabidopsis *TDF1* studies, the *attdf1* mutant showed extensive increased tapetal cell divisions (Zhu *et al*., 2008; Wu *et al*., 2023). We therefore counted tapetal cell numbers on independent transverse sections in the *hvtdf1‐2* mutant and the WT. Tapetal cell number in the *hvtdf1* mutant showed only a slight increase compared with the WT (*P* < 0.05) at the Meiosis I to Microspore release stage (Fig. S4).

**Fig. 5:**
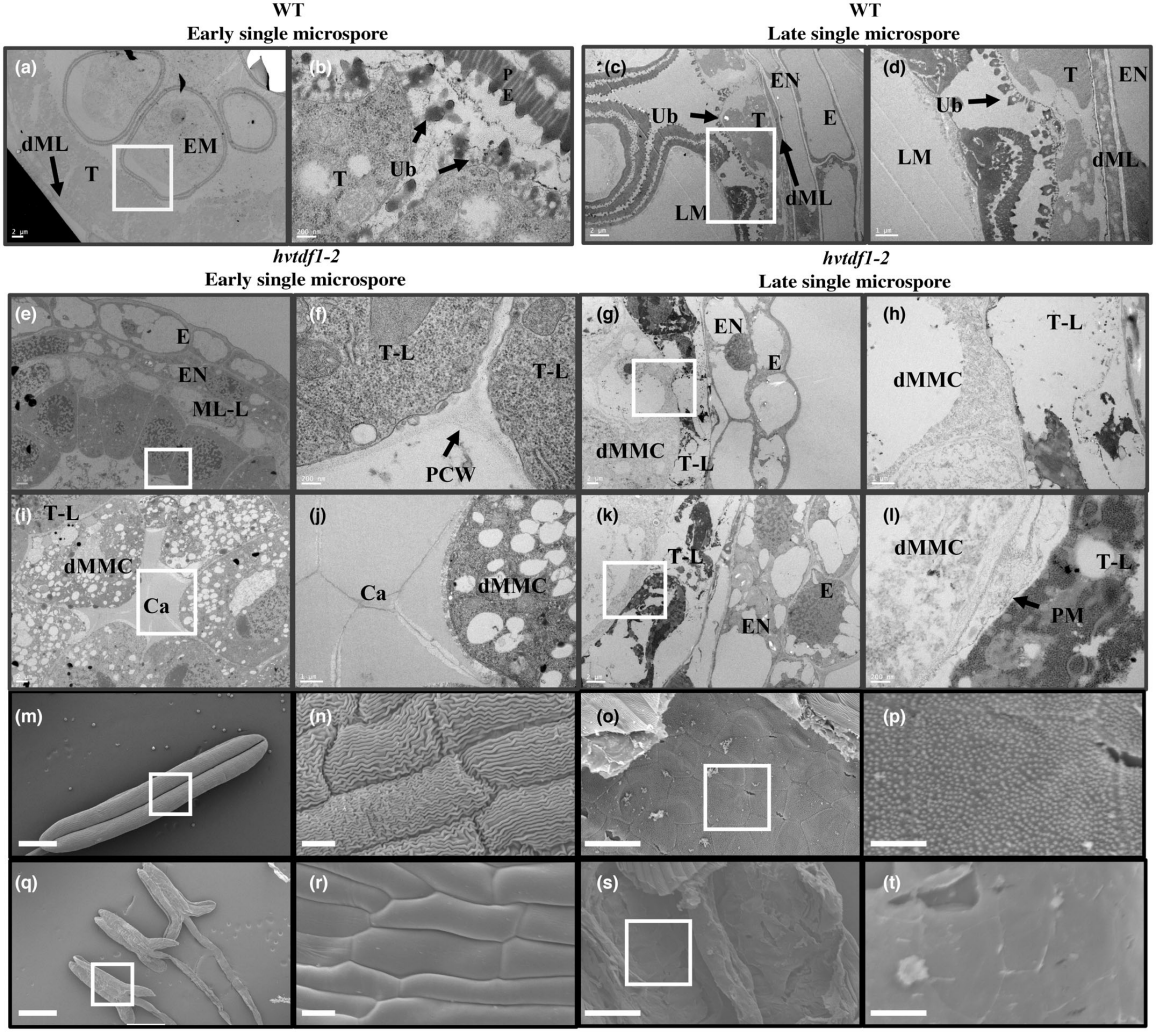
Transmission electron microscopy (TEM) and scanning electron microscopy (SEM) analysis of wild‐type and *hvtdf1‐2* anthers. TEM of (a–d) WT (e–l) *Hordeum vulgare tdf1‐2* anthers at early single microspore stage (a, b, e, f, i, j) and late single microspore stage (c, d, g, h, k, l), (b, d, f, h, j, l) are the higher magnification of the boxed regions of (a, c, e, g, i, k), respectively. SEM of (m, n) WT and (q, r) *hvtdf1‐2* anthers, and inner surface of (o, p) WT and (s, t) *hvtdf1‐2* anthers. Panels (n, p, r, t) are higher magnification of boxed regions of (m, o, q, s) respectively. Ca, callose; dML, degenerating middle layer; dMMC, defective microspore mother cell; E, epidermis; EM, early microspore; EN, endothecium; LM, late microspore; ML, middle layer; ML‐L, middle‐layer like; PCW, primary cell wall; PE, pollen exine; PM, plasma membrane; T, tapetum; T‐L, tapetum like; Ub, Ubisch body. Bars: (a, c, e, g, i, k) 2 μm; (b, f, l) 200 nm; (d, h, j) 1 μm; (m, q) 500 μm; (n, r) 10 μm; (o, s) 50 μm; (p, t) 10 μm.

To further characterize the defects in the mutant plants, transmission electron microscopy (TEM) was conducted on WT and mutant anthers. Transmission electron microscopy showed that in WT Ubisch bodies developed on the inner surface of the tapetum layer with the degenerated primary cell wall during early single microspore stage (Fig. 5b). By contrast, the primary cell wall remained in the mutants and subsequently degenerated at late single microspore stage (Fig. 5f,h). No Ubisch bodies were observed in the mutants; anthers exhibited vacuolated tapetal cells (Fig. 5g,k) and defective MMCs without exine formation (Fig. 5h,l). This suggests defects relating to sporopollenin synthesis. The SEM results also showed obvious differences between the inner and outer anther wall surfaces between WT and mutant lines. The outer surface of the WT anther has a nano‐ridge pattern and Ubisch body formation on the inner surface (Fig. 5n,p). However, a smooth pattern was seen on both the inner and outer surface of the mutant anthers (Fig. 5r,t). This suggests that HvTDF1 acts as a key regulator in tapetum development that is involved in Ubisch body formation, which is critical for pollen exine formation.

In both semi‐thin and TEM results, darker stained materials accumulated in the mutant anther locules. Previous studies in Arabidopsis and rice reported that the *tdf1* mutant showed defects in callose degeneration, which led to irregular callose accumulation in the anther locule (Zhu *et al*., 2008; Cai *et al*., 2015). We therefore conducted aniline blue staining to determine whether this material was due to a lack of callose degeneration. The results showed that in WT, callose surrounded the tetrads at stage 8b and then appeared to reduce in the newly released single microspore cells by stage 9 (Fig. S5a,b). However, in the mutant, callose remained in the central region of the anther locule rather than around the microspores at stage 8b and surrounded the defective MMCs (Fig. S5c), and this disordered accumulation appeared to increase further at stage 9 (Fig. S5d).

### Functional conserved role of *HvTDF1* in anther development

**Fig. 6:**
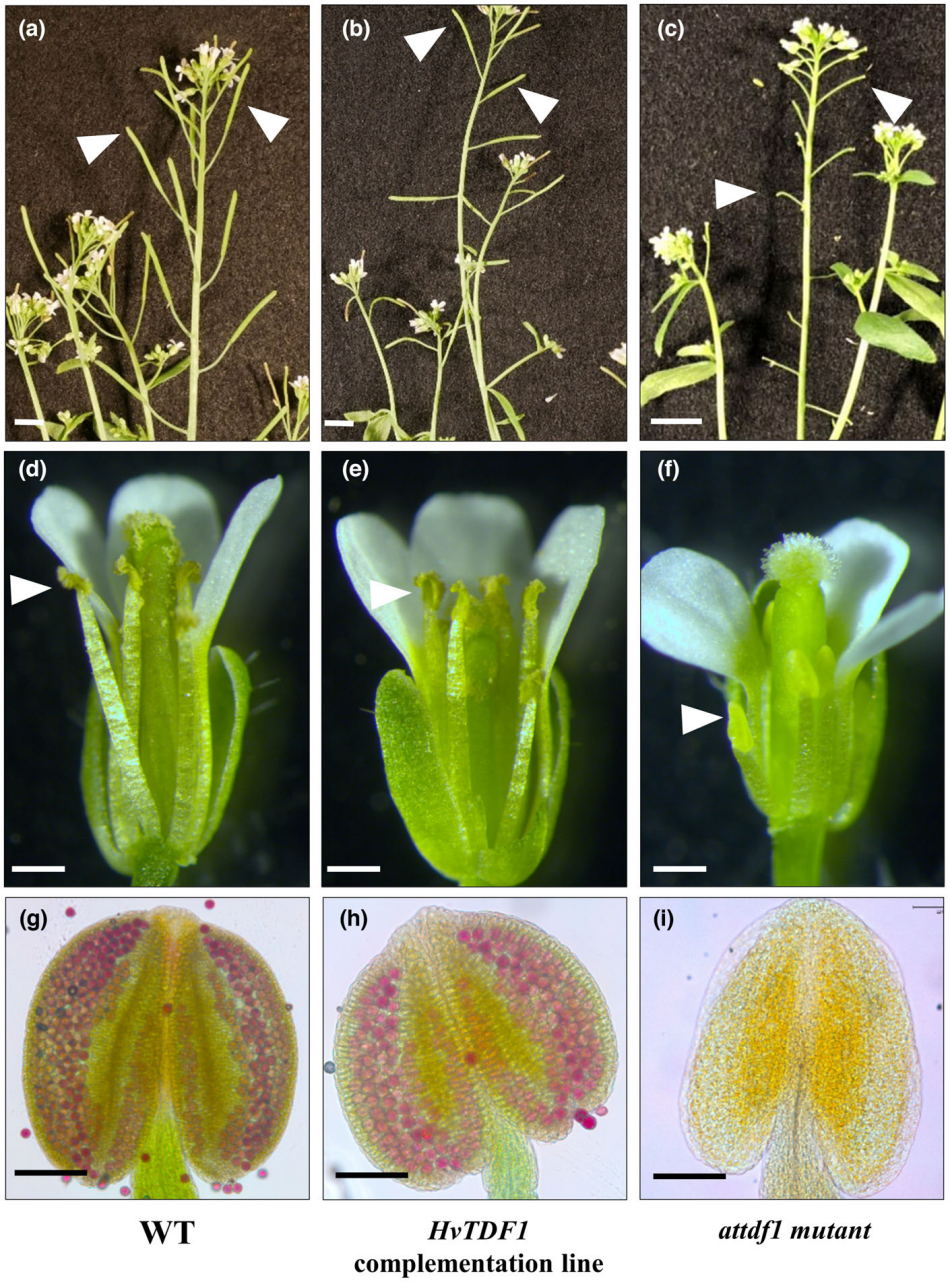
Recovery of Arabidopsis *tdf1* mutant fertility by complementation with the barley *HvTDF1* gene. *Arabidopsis thaliana* wild‐type L*er* (a, d, g), complemented Arabidopsis *tdf1* with *Hordeum vulgare TDF1* (b, e, h) and the *attdf1* mutant (c, f, i). (a–c) Arabidopsis inflorescence showing siliques (white arrowheads), (d–f) flower morphology with white arrowheads showing anthers and (g–i) Alexander pollen viability staining of anthers. Fertility is recovered in the complemented line as observed by elongated, filled siliques (b), anthers dehiscence (e) and viable pollen (magenta by Alexander staining) in anthers (h). Bars: (a–c) 50 mm; (d–f) 0.5 mm; (g–i) 100 μm.

To determine whether the putative *HvTDF1* gene has a conserved role in tapetum development, barley *HvTDF1* was tested for ability to rescue fertility in the Arabidopsis *tdf1* mutant. A plasmid carrying the native Arabidopsis *TDF1* promoter (1.23 kb) fused with the *HvTDF1* coding sequence region was introduced into the Arabidopsis *TDF1* ± heterozygous mutant. Eight independent transgenic lines were identified; two were confirmed as the homozygous mutant background, and the rest were heterozygous. Seeds were collected from these plants and resown for further screening for transgenic positive and mutant background in the next generation (Fig. S2i). The homozygous Arabidopsis *tdf1* mutant plants carrying the *HvTDF1* gene showed recovered fertility as evidenced by elongated siliques, fully formed dehiscent anthers and viable pollen (Fig. 6b,e,h).

### Transcriptome dynamic changes during the barley spike development

To help determine the biological role of HvTDF1 protein in barley, transcriptomic analysis of developmentally staged spikes, based upon anther and pollen development stages, was conducted by RNA‐sequencing. Alongside this, detailed transcriptomic analysis of WT spikes was conducted to enable comparisons to the normal regulatory process occurring during barley pollen development. Wild‐type samples were collected from five distinct anther stages, stage 6–8b. Previous studies reported that several conserved genes between rice and Arabidopsis showed specific expression patterns in anthers from stage 6 to 8b. The expression patterns of these key genes were used to confirm staging, sampling accuracy and repeatability (Fig. S6).

**Fig. 7:**
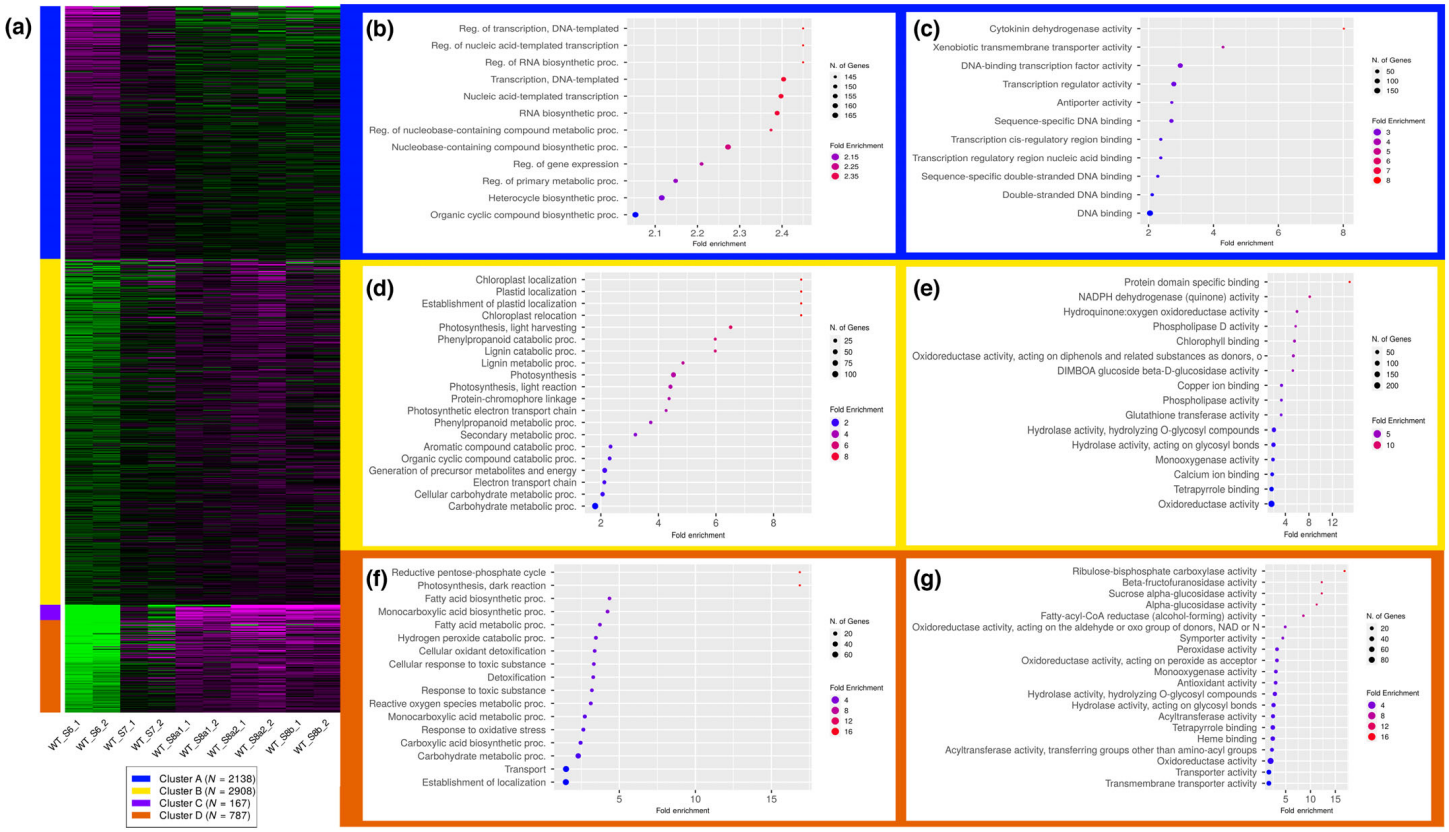
*K*‐means clustering and gene ontology (GO) term enrichment analysis of barley wild‐type spikes from stage 6 to 8b. (a) The heatmap of four clusters from top 6000 ranked genes (magenta and green colour representing the activated and repressed pattern, respectively). (b, d, f) GO term enrichment of biological process of genes and (c, e, g) GO term enrichment of molecular function of genes from cluster A (b, c), B (d–e), and D (f, g).

Principal component analysis (PCA), top 75% genes correlation matrix and hierarchical clustering heatmap analysis showed good correlation of the biological replicates from each stage (Fig. S7a–c). First, we ranked the genes for each WT sample based on standard deviation of expression, and the top 6000 genes were grouped into four different clusters by *K*‐means clustering (Fig. 7a). These genes showed specific expression patterns over spike development, such as genes in Cluster A showed expression from stage 6 to 7 and those in Cluster B showed increasing expression from stage 7 to 8a2, but decreased at stage 8b (Fig. 7a).

We performed GO term analysis for genes from each cluster; biological processes related to the transcription process were enriched in Cluster A, with many transcription factors included (Fig. 7b,c). The expression pattern of Cluster A genes, stage 6–7, suggests the spikes probably require these genes and their regulated gene network to prepare for the subsequent developmental stages. Genes from Cluster B started expression at stage 7 and peaked at 8a2, then decreased at stage 8b (Fig. 7a). GO term analysis of B cluster genes indicated that they take part in several key biological processes, such as lignin, phenylpropanoid metabolism and exhibited functions in oxidoreductase activity and ion binding (Fig. 7d,e). Cluster C identified genes involved in pollen development and pollen exine formation with some playing a molecular function in RNA glycosylase activity (Fig. S8a,b). In Cluster D, most enriched pathways related to fatty acid biosynthetic and hydrogen peroxide catabolic activity. They play key molecular functions including glucosidase activity, fatty‐acyl‐CoA reductase and peroxidase activity (Fig. 7f,g). This suggests that active metabolism processes and oxygen metabolic pathway are required for spike development. Clusters B and D genes are also associated with cellular component development, such as photosystem and components of the plasma membrane, respectively (Fig. S8c,d).

### Network analysis of *HvTDF1* in barley anther development

**Fig. 8:**
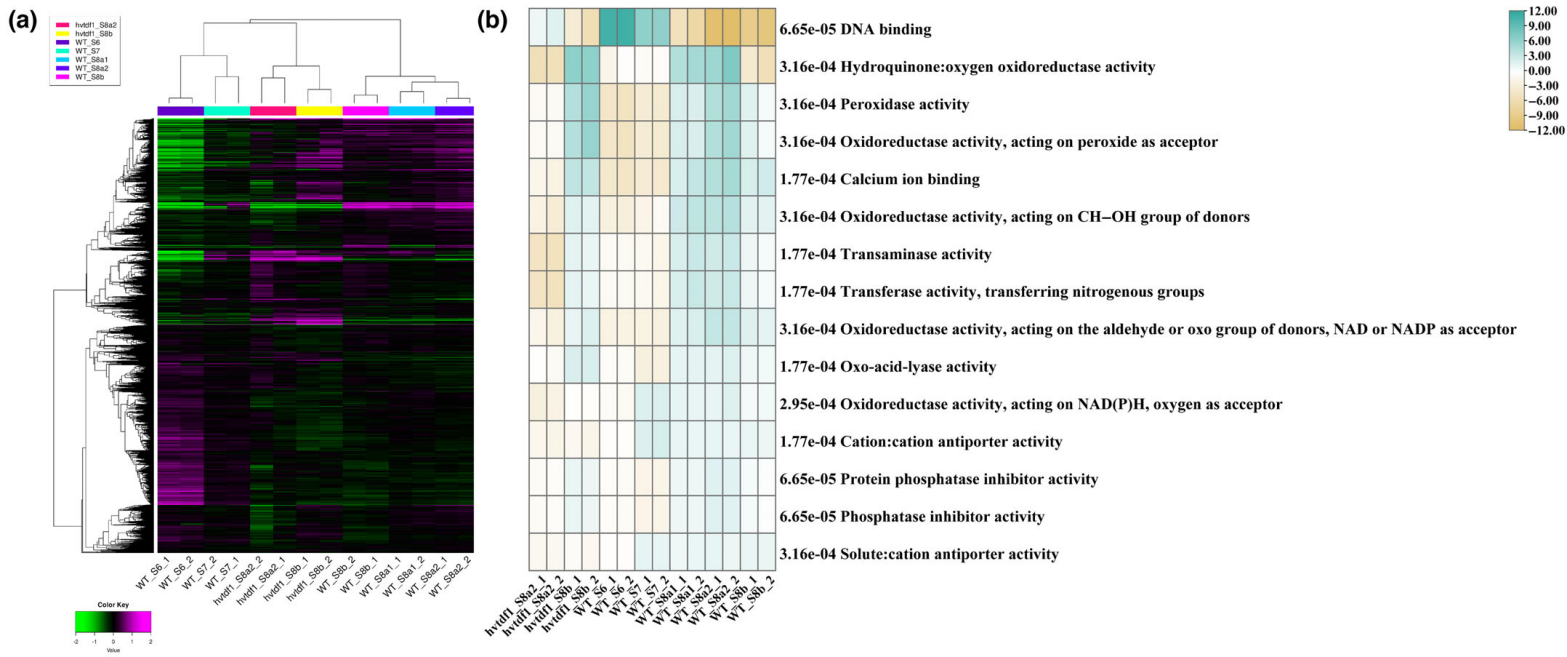
Transcriptome comparison between barley wild‐type and *hvtdf1* mutant. (a) The heatmap of hierarchical cluster conducted with standard deviation ranked top 12 000 genes across all samples. (b) The heatmap result of molecular function group conducted with PGESA package (green and yellow colour representing the activated and repressed pattern based on analysis data, respectively).

To understand the biological function of HvTDF1 in barley anther development, we conducted a comparison of transcriptome data between the *hvtdf1* mutant and WT. Spikes from the *hvtdf1‐2* mutant were collected from stage 8a2 to 8b with two biological replicates. Sample collection was based on the anther defects observed from *hvtdf1‐2* microscopy. The correlation matrix and PCA showed a good correlation between each biological replicate (Fig. S9). Interestingly, *hvtdf1* stage 8a2 and 8b had a closer correlation with the WT stage 7 data rather than the later stages (Fig. S9a). The hierarchical cluster analysis also indicated that although distinct (Fig. 8a), these results correspond to the delayed development phenotype observed in the mutant through anther semi‐thin sectioning (Fig. 4g–i).

Parametric gene set enrichment analysis (PGSEA) was used to understand the overall significant biological changes between WT and the *hvtdf1‐2* mutant. The results exhibited specific expression patterns for each process (Figs 8b, S10). In molecular function ontology, most processes relating to modulating the ROS homeostatic status were enriched, such as Hydroquinone: oxygen oxidoreductase activity (Fig. 8b). The development process in the *hvtdf1* mutant appears delayed by almost one stage compared with WT; this delay is supported by both the morphology and transcriptome data.

Based on the heatmap of enriched pathways, some of them showed delayed expression rather than downregulation; given that we have shown TDF1 to be a transcriptional activator (Fig. 2m), this suggests that these pathways are not directly regulated by HvTDF1. Therefore, the genetic pathways and genes that show downregulated in both developmental stages are potential direct targets. Such as several enriched pathways relating to the oxidoreductase activity, but showed variant molecular functions (Fig. 8b). For example, genes enriched in oxidoreductase acting in the NAD(P)H pathway exhibited downregulation in both *hvtdf1* mutant stages, and this was further confirmed by gene heatmap analysis (Fig. S11a). The other pathways, such as genes involved in cation : cation antiporter activity and pollen wall formation, also showed downregulated in *hvtdf1* (Fig. S11b,c).

**Fig. 9:**
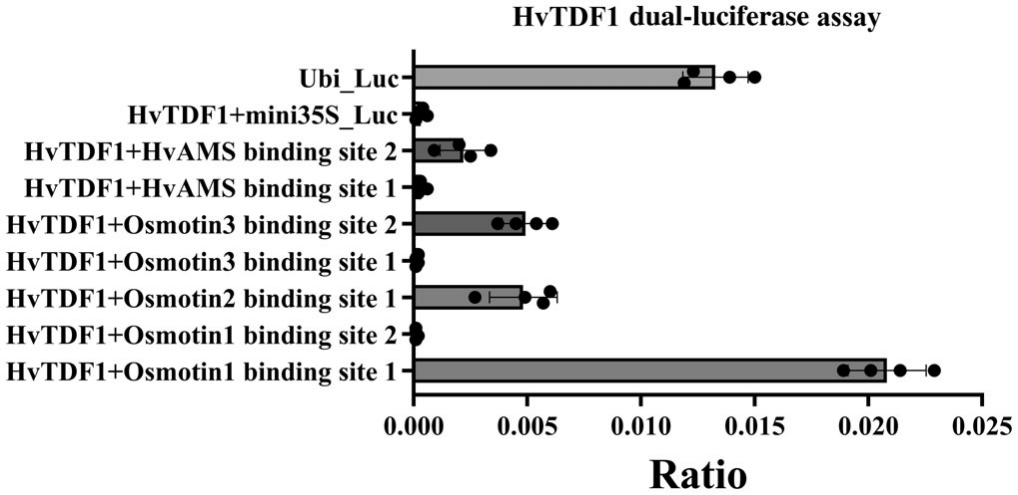
Ratio of dual‐luciferase assay results of HvTDF1 and putative regulating targets. Modified dual‐luciferase reporter (DLR) transcription factor activation assay of *Hordeum vulgare* TDF1, on putative targets HvAMS, Osmotin1/2/3. The transaction level of positive (Ubi_Luc), HvTDF1 and its putative targets, and a negative effector (mini35S_Luc) presented as firefly : Renilla LUC ratio, which equals to the measured firefly luciferase luminescence normalised by the luminescence of the constitutively expressed Renilla luciferase. Luc, luciferase; mini35S, Mini Cauliflower Mosaic Virus 35S promoter; Ubi, Arabidopsis Ubiquitin 10 promoter. Error bar is the SE of four biological replicates.

To identify potential direct targets for HvTDF1, we used a more restrictive screening filter (*q* < 0.05 and fold change < −4), which identified 57 genes. We further performed Blast to identify their orthologues genes in Arabidopsis to help address their biological functions (Table S3). The barley putative orthologues genes include *HvCYP704B1*, *HvQRT2*, and *HvACOS5* (Fig. S12). However, previous studies in Arabidopsis showed that these genes were directly regulated by downstream AMS/MYB80 rather than TDF1 (Wang *et al*., 2018). Three osmotin protein family genes were included in the putative HvTDF1 direct target list as identified by differential expression in *hvtdf1‐2* (Table S3). In WT, they had a similar RNA expression pattern as *HvTDF1*, suggesting a potential direct regulatory relationship with HvTDF1 (Fig. S13). Dual‐luciferase assay confirmed direct HvTDF1 activation of these genes (Fig. 9). In Arabidopsis, AMS is the direct target of TDF1, the barley orthologous gene was therefore also tested and *HvAMS* was shown to be activated by HvTDF1 (Fig. 9).

### Dissecting the biological role of TDF1 genes in anther development in monocot and dicot plants through transcriptome comparison

The HvTDF1 protein can rescue the fertility of the Arabidopsis *tdf1* mutant, and as TDF1 can directly activate *AMS*, this suggests that these two proteins share conserved functions. However, whether their role in controlling anther and pollen development, in terms of network regulation and biological processes, is similar in monocot and dicot plants remains unknown. Therefore, we tried to answer this question through comparison of the transcriptome changes, particularly downregulated genes (*q* < 0.05 and Fold change < −1), between the barley and Arabidopsis *tdf1* mutants (Tables [Link], [Link]).

**Fig. 10:**
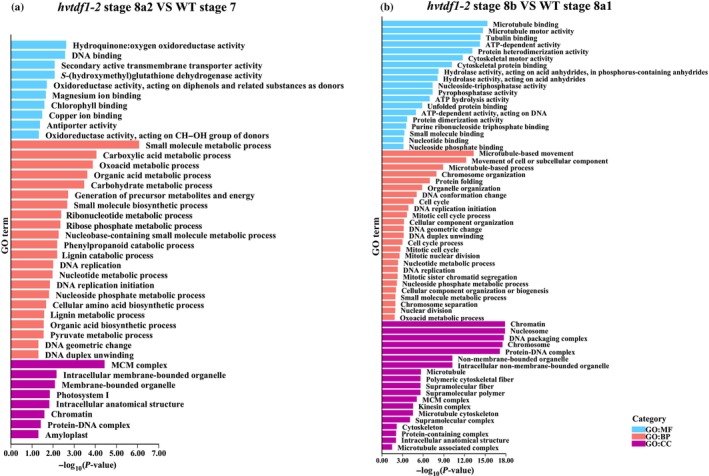
Transcriptome comparison between the downregulated genes from *hvtdf1* two stages. (a) Gene ontology (GO) term analysis the genes downregulated from *Hordeum vulgare tdf1* mutant from stage 8a2 vs wild‐type stage 7. (b) GO term analysis the genes from down‐regulated in *hvtdf1* mutant from stage 8b vs wild‐type stage 8a1. BP, biological process; CC, cellular component; GO, gene ontology category; MF, molecular function.

We performed GO term enrichment analysis with the downregulated genes from barley and Arabidopsis, respectively (Figs 10, S14, S15). For barley, we first performed two comparison groups, *hvtdf1* stage 8a2 vs WT stage 7, and *hvtdf1* stage 8b vs WT stage 8a1. These combinations were selected based on the delayed development between WT and mutant plants. The *attdf1* transcriptome data were extracted from previously published microarray data (Li *et al*., 2017). Both barley combinations showed that key genes related to the microtubule movement, DNA replication and cell cycle were downregulated (Fig. 10; Tables [Link], [Link]). For the ontology of cellular components, the enrichments were minichromosome maintenance (MCM) protein complex, DNA packaging complex, cytoskeleton development related (Fig. 10; Tables [Link], [Link]). These results may explain the observed delayed endomitosis and irregular meiosis phenotype (Fig. 4h–j). However, in Arabidopsis *tdf1* mutant microarray data, GO term enrichment showed the ontology of biological process related to pollen development, pollen exine formation and sporopollenin biosynthetic process (Fig. S14; Table S7). The ontology of cellular component showed the enrichment of plasma membrane component, pollen coat and pollen tube (Fig. S14; Table S7). The GO term analysis showed no relationship between each species. Thus, we looked at the sampling method used for the *attdf1* microarray analysis, Arabidopsis bud samples included buds with various development stages of anthers, which may result in bias and background noise. We then performed the comparison between *hvtdf1*‐2 stage 8b vs WT stage 8b. The GO term enrichment results were similar to *attdf1*, such as the ontology of biological process, the processes relating to pollen exine formation, pollen wall formation and gametophyte development and sporopollenin biosynthetic process were enriched. The ontology of cellular component still showed some pathways that relating to DNA packaging as mentioned previously (Fig. S15; Table S8).

A further Blast analysis allowed identification of barley and Arabidopsis orthologues to allow gene comparisons, of the 931 *attdf1* downregulated genes only 99 genes were also only downregulated in *hvtdf1*, and these included *AMS*, *ACOS5*, *MS2*, *LAP5/6*, *MYB80*, *KCS7/21*, and *SHT*. Gene ontology term analysis showed enrichment in pollen development, sporopollenin biosynthetic process and pollen exine formation, highlighting the shared role that both these proteins play in controlling pollen development (Table S9). Five hundred and forty‐four genes were not differentially expressed in *hvtdf1*, the GO term analysis showed pathways involved in later pollen development stages such as pollination and pollen tube growth (Table S10) and this highlights the difference in staging between the two datasets rather than a separate function within the two species.

*ABORTED MICROSPORES* and *MYB80*/MS188, are downstream genes of TDF1 in both rice and Arabidopsis (Sorensen *et al*., 2003; Xu *et al*., 2010; Phan *et al*., 2011; Li *et al*., 2017). Their putative barley orthologous genes also showed downregulated in *hvtdf1* (Fig. S16a,b). However, some AMS expression was retained in the *hvtdf1* mutant (Fig. S16a). Based on the expression pattern of *AMS*, which was earlier than *TDF1* in stage 6 (Fig. S6f), these results suggest that TDF1 is involved in regulating *AMS* expression, but that this is not exclusive, with induction also occurring by additional factor(s) during anther development. The consistent high expression level of AMS from the anther development stage 7–8b is critical for normal anther development (Ferguson *et al*., 2017), and TDF1 may play an important role maintaining it to allow pollen development, but does not have sole regulation as observed in Arabidopsis.

## Discussion

### *TDF1* genes may be conserved in both monocot and dicot plants

With the increasing population size and climate changes, increasing environmentally sustainable food production is needed. However, a lack of genetic resources and understanding of crop reproduction is limiting the application of one potential tool to address this, that of hybrid vigour (Govindaraj *et al*., 2015). In this study, we characterize HvTDF1, an R2R3 MYB family transcription factor involved in controlling tapetum development in the monocot crop barley. This is only the second male sterility gene that has been characterized in barley and as such offers a potential breeding resource for generating hybrid barley. We adopted a reverse genetic approach to identify and characterize HvTDF1, using the previously characterized AtTDF1, OsTDF1 and putative BdTDF1 sequences for comparison against the barley database and identified the putative *HvTDF1* orthologous gene (Zhu *et al*., 2008; Cai *et al*., 2015; Zhang *et al*., 2019). RT‐qPCR and RNA *in situ* hybridization were used to help confirm gene identity. *HvTDF1* was specifically expressed during pollen development in the tapetum, starting from anther stage 7 and peaking at the tetrad stage (Fig. 2a–d).

Cai *et al*. (2015) showed that the rice OsTDF1 could recover the fertility of the Arabidopsis *tdf1* mutant, suggesting a potentially conserved role for the *TDF1* genes in monocot and dicot plants. Our complementation analysis using the *HvTDF1* gene recovered fertility of Arabidopsis *tdf1* mutant further confirming the conserved role of *TDF1* genes in monocot and dicots (Fig. 6b,e,h). Accurate staging systems for reproductive development are needed to help understand the developmental process and the defects between WT and mutant lines. We first found that mutation of the *HvTDF1* gene did not affect the size of the barley spike, with the spikes being of comparable sizes for the same stages. However, comparison between equivalently sized samples detected different speeds of development in the mutant. To identify the precise defects of *hvtdf1*, we collected spikes every 0.1 cm from the barley inflorescence initiation stage from WT and mutant. Both WT and mutant showed equivalent progression of anther development until stage 7 (Fig. 4a,f); however, when the WT finished the meiosis process, *hvtdf1* had not completed meiosis I (Fig. 4c,h). This delayed development in *hvtdf1* suggests a critical role for HvTDF1 in regulating the progression of tapetum development. Ectopic expression of *AtTDF1* regulated by the promoter of the upstream transcription factor DYT1 was unable to recover fertility in the *dyt1* mutant (Gu *et al*., 2014). This further confirms the need for precise spatial and temporal expression of the *TDF1* gene for its normal function.

Our results indicate that two key features of tapetum development, cell endomitosis and cell wall degeneration, that occur during stage 8a and 9 in WT are delayed in *hvtdf1* (Figs 4i, 5f). This suggests that although tapetum cells in *hvtdf1* acquire tapetum cell fate, their normal developmental progression cannot occur without HvTDF1. Tapetum cell fate determination was reported as regulated by the conserved gatekeeper, DYT1 (Zhang *et al*., 2006), which has also been characterized in rice and maize (Jung *et al*., 2005; Moon *et al*., 2013). Phenotyping results from *dyt1* ortholog mutants indicates that the irregular tapetum development, with tapetum cells still showing periclinal divisional ability, suggesting the tapetum layer fails to acquire cell fate determination (Jung *et al*., 2005; Moon *et al*., 2013). In our RNA‐seq data, the expression level of the barley *HvDYT1* ortholog gene was upregulated in the *hvtdf1* mutant (Fig. S16c). Whereas the putative orthologs of the TDF1 downstream genes, AMS and MYB80, were downregulated (Fig. S16a,b), these have been reported as involved in mature tapetum development, such as exine formation and callose degeneration (Phan *et al*., 2011; Li *et al*., 2017; Lou *et al*., 2018; Wang *et al*., 2018; Lu *et al*., 2020). These results suggest that the cell fate of the tapetum is determined by upstream genes, such as DYT1, but the subsequent development, such as endomitosis, and tapetum cell wall degeneration may be specifically regulated by TDF1, and that the later development process may be regulated by other key transcription factors. These results also suggest conservation of the tapetum regulatory pathway in barley.

### *HvTDF1* may regulate the rate of tapetum development through osmotin proteins

Previous studies identified the direct regulatory relationship between these five conserved genes, *DYT1‐TDF1‐AMS‐MYB80‐MS1*, which play critical roles in tapetum development (Gu *et al*., 2014; Xiong *et al*., 2016; Lou *et al*., 2018; Lu *et al*., 2020). Comparison of microarray data from these five mutants helped provide understanding of the regulatory roles (Li *et al*., 2017). The comparison of gene lists of the twofold downregulated between Arabidopsis mutants of *TDF1* and its upstream gene, *DYT1*, and downstream gene, *AMS*, showed that *TDF1* mainly focusses on controlling reactive oxygen molecules, such as ROS, or other oxidase species, within the tapetum. These have been confirmed as involved in the programmed cell death process, or as the signalling molecules triggering downstream gene expression in Arabidopsis (Xing & Zachgo, 2008; Xie *et al*., 2014; Li *et al*., 2017).

The modified dual‐luciferase assay indicates that HvTDF1 protein can independently activate gene expression *in vivo* (Fig. 2m); however, our RNA‐seq data indicate that most pathways in *hvtdf1* showed delayed expression rather than downregulation, which also corresponds to the phenotype we observed from anther sections (Figs 4g,h, 8b, S10). The pathways downregulated in both stage 8a2 and 8b may be the directly regulated pathway of HvTDF1, which include oxidoreductase activity, acting on NAD(P)H, cation : cation antiporter (Fig. 8b). Potential direct targets of HvTDF1 were identified from the RNA‐seq analysis and indicated some common downstream genes such as *CYP704B1*, *QRT2*, and *ACOS5*, which are direct targets of Arabidopsis AMS and MYB80 (Fig. S12; Wang *et al*., 2018). These results suggest that the downstream pathways of TDF1 share conserved parts between barley and Arabidopsis. Three osmotin genes, *Osmotin1/2/3*, were identified from the screening and showed similar WT expression patterns as *HvTDF1*, but almost no expression in the *hvtdf1‐2* mutant (Fig. S13a–c). Dual‐luciferase assay results indicate that HvTDF1 directly binds to the promoter regions and activates these genes, with strongest transactivation of the *Osmotin1* gene (Figs 9, S13a).

The function of osmotin proteins in anther development has not been reported; however, previous studies indicate that osmotin proteins show distinct developmental and organ specific expression, suggesting they play different roles in various developmental processes (Kononowicz *et al*., 1992). They have been linked to osmotic stress tolerance through accumulation of the osmolyte proline and the associated quenching of reactive oxygen species and free radicals (Kumar *et al*., 2015). During anther development, both somatic and germline cell layers show specific dynamic hypoxia and ROS production in each cell layer (Yu & Zhang, 2019). The ROS levels of each cell type are important for signalling transduction and trigger normal development processes, such as cell differentiation, proliferation and PCD (Yu & Zhang, 2019). At anther development stage 5, all four somatic cell layers are established, with the middle layer showing the highest ROS accumulation levels and least accumulation in the tapetum layer. When anther meiosis initiates, ROS accumulation starts in tapetum layer from stage 7 to 9, which corresponds to the specific expression patterns of the oxidoreductase and peroxidase genes in our WT RNAseq data (Fig. 8b). However, in *hvtdf1* mutant, these genes showed delayed expression patterns (Fig. 8b). Studies have indicated a relationship between osmotin and ROS accumulation, with osmotin detoxifying ROS and free radicals during osmotic stress by modulating proline accumulation (Floyd & Nagy, 1984; Lutts *et al*., 1996; Hong *et al*., 2000; Vinocur & Altman, 2005; Kumar *et al*., 2015). Osmotin overexpression plants have higher activities of ascorbate peroxidase (APX) and superoxide dismutase (SOD), which contributed to detoxify the accumulated H_2_O_2,_ but the mechanism behind this still remains unknown (Kumar *et al*., 2015). Arabidopsis *TDF1* has previously been shown to directly target *SKEWED5‐SIMILAR 18* (*SKS18*), which has ascorbate oxidase activity and is involved in ROS homeostasis; this ROS homeostasis is proposed to modulate the switch between tapetum cell division and differentiation in Arabidopsis (Wu *et al*., 2023). One of the potential roles of HvTDF1 may be through regulation of osmotin protein expression to modulate the homeostatic status of ROS in tapetal cells to ensure normal development. The crosstalk between ROS and osmotin proteins may therefore trigger and serve to regulate the rate of downstream biological processes, such as tapetal cell nuclei endomitosis, tapetal development and PCD.

In Arabidopsis, TDF1 directly binds to a beta‐expansin family protein, EXPB5, which may play a critical role in adjusting cell turgor pressure through external cell wall modification (Lou *et al*., 2018). In WT, dark staining of the tapetum layer indicates highly active biological processes (Fig. 4a–d), with various materials generated in tapetal cells and delivered into the adjacent anther locule; this fails to occur in the *hvtdf1‐2* mutant. The solute concentration around the tapetum cells is likely to be rapidly changing and resulting in potential osmotic shock. Osmotin proteins have been shown to protect against osmotic shock during abiotic stress through compartmentalizing the solutes, or altering the metabolism or structure in cells (Singh *et al*., 1987; Barthakur *et al*., 2001; Kumar *et al*., 2015). Here, a potential role of these barley osmotin proteins in tapetum development may be via similar molecular functions to protect against the associated abiotic stress of pollen development. Under normal development, these osmotin proteins may trigger changes in tapetal cells, which protect them and ensure normal development processes under the rapid osmotic dynamic changes, potentially via quenching and moderation of hypoxia and reactive oxygen species and compartmentation of solutes. This may in turn result in changes to cell wall modifications turgor via modification of beta‐expansins.

### The TDF1 proteins share conserved, but also unique pathways in anther development

The GO term analysis of downregulated genes from two stages of the *hvtdf1* mutant show that several key biological processes have been affected in this mutant, for example, organelle development, ATP and ADP metabolism, pollen wall development (Fig. 10), which have not been previously described. The comparison between GO term analysis from barley and Arabidopsis, as well as Blast analysis for orthologue comparison, suggests that these two species share conserved pathways, such as pollen wall assembly, and sporopollenin biosynthesis (Figs 10, S14). However, some enriched downregulated pathways in Arabidopsis *tdf1* were delayed in *hvtdf1*, such as the anchored component of membrane (Figs S10, S14). This reveals the importance of accurate sampling in transcriptome analysis and indicates that barley is a good model plant to study anther development. These findings suggest that HvTDF1 might be involved in regulating these biological processes, and/or controlling the expression of other master regulators, such as AMS, which we have shown that HvTDF1 can directly activate similar to that observed in Arabidopsis (Lou *et al*., 2018).

The enrichment of highly active metabolic processes in *tdf1* also suggests that the anther status is still under developmental flux when TDF1 is expressed, since key genes relating to cell organelle development are downregulated in *hvtdf1*. The key components required for the subsequent tapetum development process may therefore be directly regulated by TDF1, or its downstream regulated genes. TDF1 and the downstream target genes appear essential for facilitating tapetum development from the newly established cell layer to the mature cell layer to perform the required biological roles for pollen grains maturation. Currently, there is significantly less information available regarding tapetum development in barley and the characterized gene network, compared to Arabidopsis or rice. The lack of the RNA‐seq data for mutants of HvTDF1 downstream gene targets, such as HvAMS, means that it is therefore difficult to extract the directly regulated genes from the RNA‐seq data of the *hvtdf1* mutant. However, our fine‐staged RNA‐seq barley anther data have made it possible to identify HvTDF1 downstream targets and characterise the regulatory network.

### TDF1 may not be the master regulator that regulates barley AMS expression

Previous studies showed that AMS is downstream of TDF1 and directly regulated by TDF1 in Arabidopsis (Zhu *et al*., 2008; Lou *et al*., 2018). In our RNA‐seq data, while *AMS* expression is reduced, it is not completely absent in the *hvtdf1* mutant (Fig. S16a). This suggests some other transcription factors may also take part in regulating *AMS* expression. Based on the expression pattern of AMS in barley, HvAMS is expressed slightly earlier than HvTDF1, indicating that AMS is also regulated by other currently unknown transcription factors (Fig. S6e,f). The dual‐luciferase assay results showed that HvTDF1 binds to the HvAMS promoter and activates the firefly luciferase reporter gene expression; however, HvTDF1 showed less activation of *HvAMS* expression compared with the other osmotin proteins (Fig. 9). In Arabidopsis, *MYB80*/*MS188* is downstream of *AMS*, which was shown to be directly regulated by an AMS–TDF1 complex (Lou *et al*., 2018). In Arabidopsis, the luciferase reporter assay showed that either AMS or TDF1 could bind to the MYB80 promoter and activate expression; however, expression was dramatically increased when both TDF1 and AMS were co‐expressed, suggesting the important role of TDF1–AMS complex in regulating MYB80/MS188 expression (Lou *et al*., 2018). In our RNA‐seq data, the barley MYB80 orthologous gene was not expressed (Fig. S16b), suggesting both HvTDF1 and HvAMS are needed to activate HvMYB80. In agreement, in the *atams* mutant, the expression level of *TDF1*, as the upstream gene of AMS, was not affected, but MYB80 still was not expressed. These results suggest that TDF1 and AMS are unable to work alone in regulating MYB80 in these species.

All these findings indicate that the gene network between TDF1 and AMS is not as simple as an inclusion relationship. Each of them takes responsibility to regulate the key anther developmental processes. The network of each gene appears to overlap; however, they have different roles in determining tapetum development, from initiation to maturation, then programmed cell death to release of materials for pollen wall and pollen coat development.

## Competing interests

None declared.

## Author contributions

ZAW led the project. ZAW, JFG, WY and MH designed the experiments. MH, WY, JFG and SS performed the experiments. MH, AT, GX and JZ performed the transcriptome data analysis with barley and Arabidopsis data. ZAW, MH, AT and WY wrote, discussed and edited the paper. MH and WY contributed equally to this work. The author responsible for distribution of materials integral to the findings presented in this article is ZAW (zoe.wilson@nottingham.ac.uk).

## Supporting information


**Fig. S1** Confirmation of CRISPR/Cas9 *TDF1* mutants.
**Fig. S2** Characterization of barley *tdf1* mutants in cv Golden Promise.
**Fig. S3** Meiosis process in wild‐type and *hvtdf1‐2* male meiocytes.
**Fig. S4** Tapetal cell number between wild‐type and *hvtdf1‐2*.
**Fig. S5** Analysis of callose distribution in wild‐type and *hvtdf1‐2* anthers.
**Fig. S6** RNA‐seq analysis of barley tapetum transcription factors in wild‐type.
**Fig. S7** Correlation and distance between different wild‐type RNA‐seq samples.
**Fig. S8** Gene ontology term analysis results of different gene sets from *K*‐means clusters.
**Fig. S9** Principal component analysis and correlation matrix analysis results between wild‐type and *hvtdf1*.
**Fig. S10** Heatmap of biological process and cellular component groups.
**Fig. S11** Heatmap of gene expression pattern from PGSEA analysis results.
**Fig. S12** RNA‐seq expression analysis of orthologous TDF1 downstream genes.
**Fig. S13** RNA‐seq expression analysis of osmotin proteins.
**Fig. S14** Gene ontology term analysis of the downregulated genes from *attdf1* microarray data.
**Fig. S15** Gene ontology term analysis of the downregulated genes from stage 8b.
**Fig. S16** Expression pattern of putative barley orthologous genes.

**Table S1** List of primers used.

**Table S2** Barley DEGS with *P* = 0.05 and log_2_FC −4.

**Table S3** Barley DEGs between wild‐type and *hvtdf1*.

**Table S4** Analysed *attdf1* microarray data and downregulated genes.

**Table S5** Gene ontology term results of *hvtdf1‐2* stage 8a2 vs wild‐type stage 7.

**Table S6** Gene ontology term results of *hvtdf1‐2* stage 8b vs wild‐type stage 8a1.

**Table S7** Gene ontology term results of *attdf1* vs wild‐type.

**Table S8** Gene ontology term results of *hvtdf1‐2* stage 8b vs wild‐type stage 8b.

**Table S9** Gene ontology term results of down DEGs from *attdf1* and *hvtdf1*.

**Table S10** Gene ontology term results of *attdf1* not *hvtdf1* down DEGs.
Please note: Wiley is not responsible for the content or functionality of any Supporting Information supplied by the authors. Any queries (other than missing material) should be directed to the *New Phytologist* Central Office.

## Data Availability

The data that support the findings of this study are available in the supplementary materials of this article. HvDYT1 (HORVU.MOREX.r2.2HG0112900), TDF1 (AT3G28470), HvTDF1 (HORVU MOREX r2.4HG0319540), AMS (AT2G16910), HvAMS (HORVU.MOREX.r2.6HG0456990), HvMYB80 (HORVU.MOREX.r2.2HG0144320), HvGAMYB (HORVU.MOREX.r2.3HG0246570), HvEMS1 (HORVU.MOREX.r2.3HG0258570), HvTDL1a (HORVU.MOREX.r2.1HG0022010), HvMS1 (HORVU.MOREX.r2.5HG0402180), Osmotin 1 (HORVU.MOREX.r2.4HG0299130), Osmotin 2 (HORVU.MOREX.r2.5HG0436790), Osmotin 3 (HORVU.MOREX.r2.5HG0416120).
