# Supplementary material for: Barley TAPETAL DEVELOPMENT and FUNCTION1 (HvTDF1) gene reveals conserved and unique roles in controlling anther tapetum development in dicot and monocot plants
Source: New Phytol. 2023 Aug 10;240(1):173–90. doi: 10.1111/nph.19161 (PMC10952600; doi:10.1111/nph.19161)
Supplement: Supplementary file 1 — Fig. S1 Confirmation of CRISPR/Cas9 TDF1 mutants. Fig. S2 Characterization of barley tdf1 mutants in cv Golden Promise. Fig. S3 Meiosis process in wild‐type and hvtdf1‐2 male meiocytes. Fig. S4 Tapetal cell number between wild‐type and hvtdf1‐2. Fig. S5 Analysis of callose distribution in wild‐type and hvtdf1‐2 anthers. Fig. S6 RNA‐seq analysis of barley tapetum transcription factors in wild‐type. Fig. S7 Correlation and distance between different wild‐type RNA‐seq samples. Fig. S8 Gene ontology term analysis results of different gene sets from K‐means clusters. Fig. S9 Principal component analysis and correlation matrix analysis results between wild‐type and hvtdf1. Fig. S10 Heatmap of biological process and cellular component groups. Fig. S11 Heatmap of gene expression pattern from PGSEA analysis results. Fig. S12 RNA‐seq expression analysis of orthologous TDF1 downstream genes. Fig. S13 RNA‐seq expression analysis of osmotin proteins. Fig. S14 Gene ontology term analysis of the downregulated genes from attdf1 microarray data. Fig. S15 Gene ontology term analysis of the downregulated genes from stage 8b. Fig. S16 Expression pattern of putative barley orthologous genes. [file NPH-240-173-s006.pdf]

New Phytologist Supporting Information
Article title: Barley *TAPETAL DEVELOPMENT* and *FUNCTION1* (*HvTDF1*) gene reveals conserved and unique roles in controlling anther tapetum development in dicot and monocot plants
Authors: Miaoyuan Hua, Wenzhe Yin, José Fernández Gómez, Alison Tidy, Guangwei Xing, Jie Zong, Shuya Shi, Zoe A. Wilson
Article acceptance date: 20 June 2023

Supplementary Figures

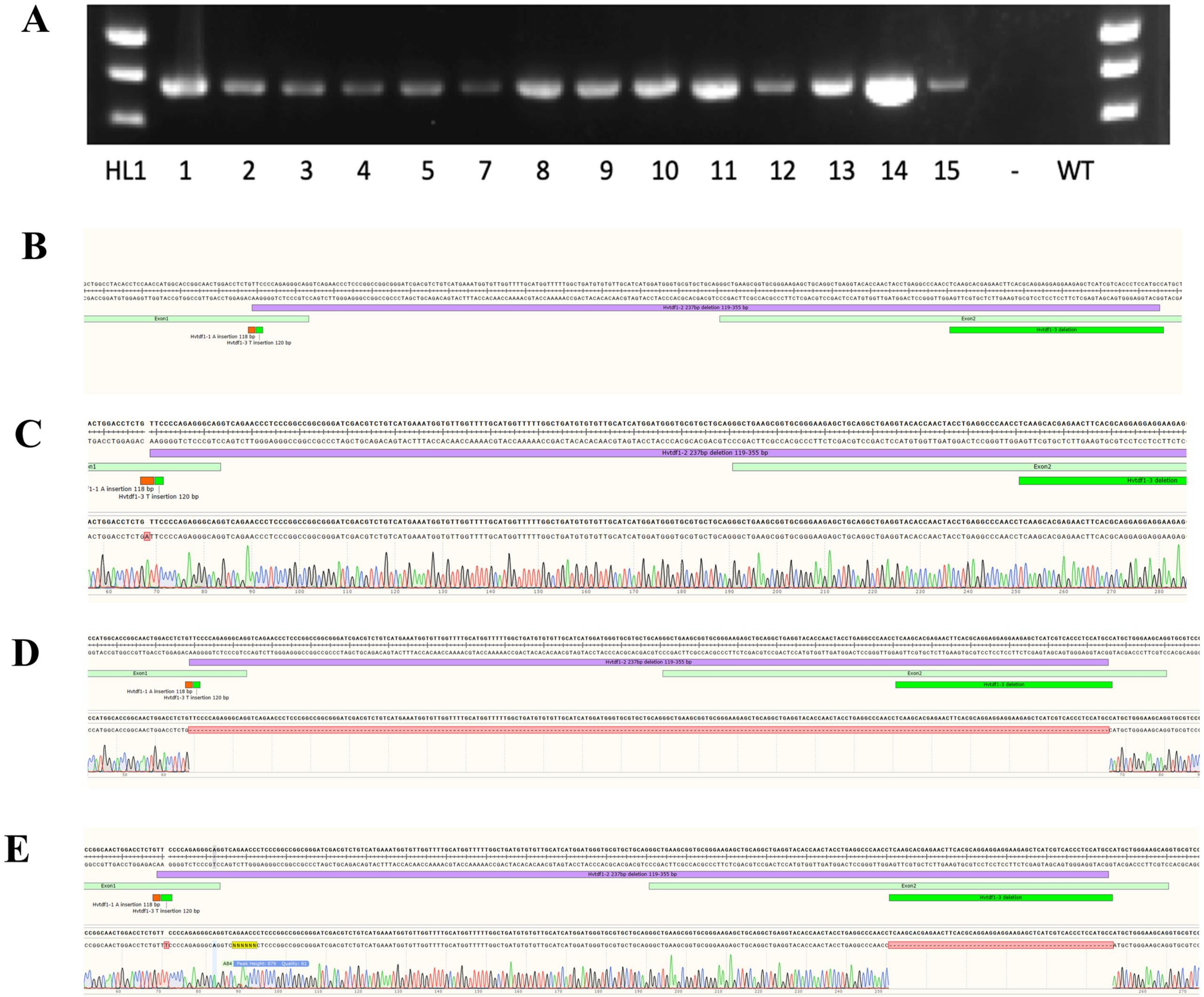

Fig. S1. Confirmation of CRISPR/Cas9 *TDF1* mutants. (A) PCR confirmed all transformants were positive using 5149/5150 primers in the T0 generation. (Amplicon size = 448 bp). (B) Diagram of location of the three independent *hvtdf1* CRISPR mutant alleles. *hvtdf1-1*: Insertion 1bp, Adenine, at 118 bp from start codon on gDNA in Orange; *hvtdf1-2*: 237 bp deletion from 119 bp to 355 bp on gDNA in purple; *hvtdf1-3*: Insertion 1bp, Thymine, at 120 bp, 56 bp deletion from 301 bp to 356 in Green. (C-E) Sequenced region of *hvtdf1* mutations, *hvtdf1-1* (C), *hvtdf1-2* (D), *hvtdf1-3* (E).

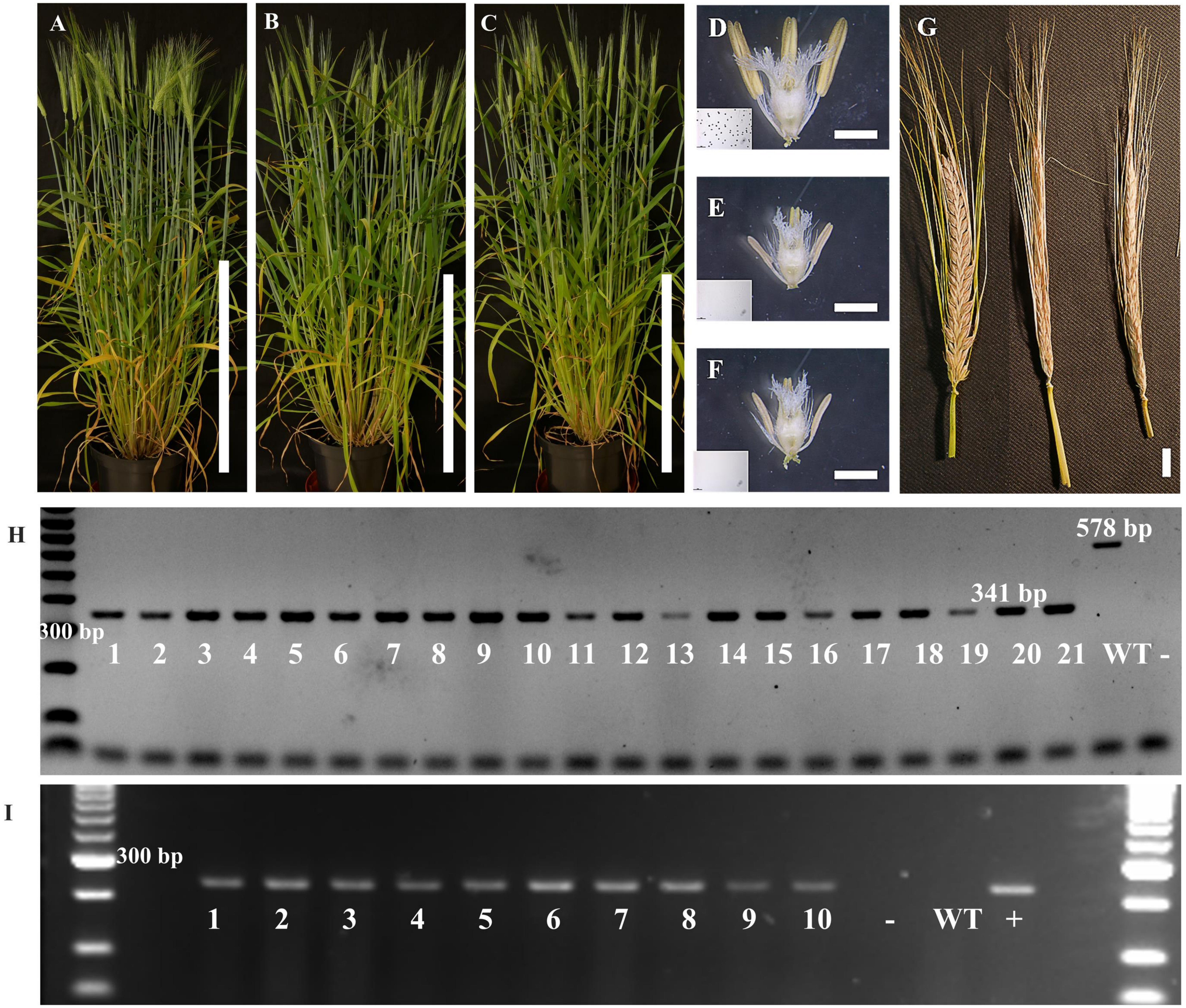

Fig. S2. Characterization of barley *tdf1* mutants in cv. Golden Promise. Comparison between whole plant morphology of (A) wild-type and (B) *hvtdf1-1* and (C) *hvtdf1-3* mutants. (D) Wild-type and (E) *hvtdf1-1* and (F) *hvtdf1-3* floral tissues with removed lemma and palea, the inserted photos are the KI-I_2 stained pollen. (G) Wild-type fertile (Left) and *hvtdf1-1*(Middle) *and hvtdf1-3* (Right) sterile mature spikes. Bar = 50cm (A-C), 1mm (D-F), 1cm (G). (H) Genotyping results of *hvtdf1-2* showing the wild-type band (578bp) and the smaller mutant band (samples 1-21; 341bp) indicative of the CRISPR mutation. (I) Genotyping results of Arabidopsis complementation line with T-DNA specific primer combinations (Line 1-10 were positive; no bands were observed in negative control and wild-type plants, the transgenic vector used as the positive control).

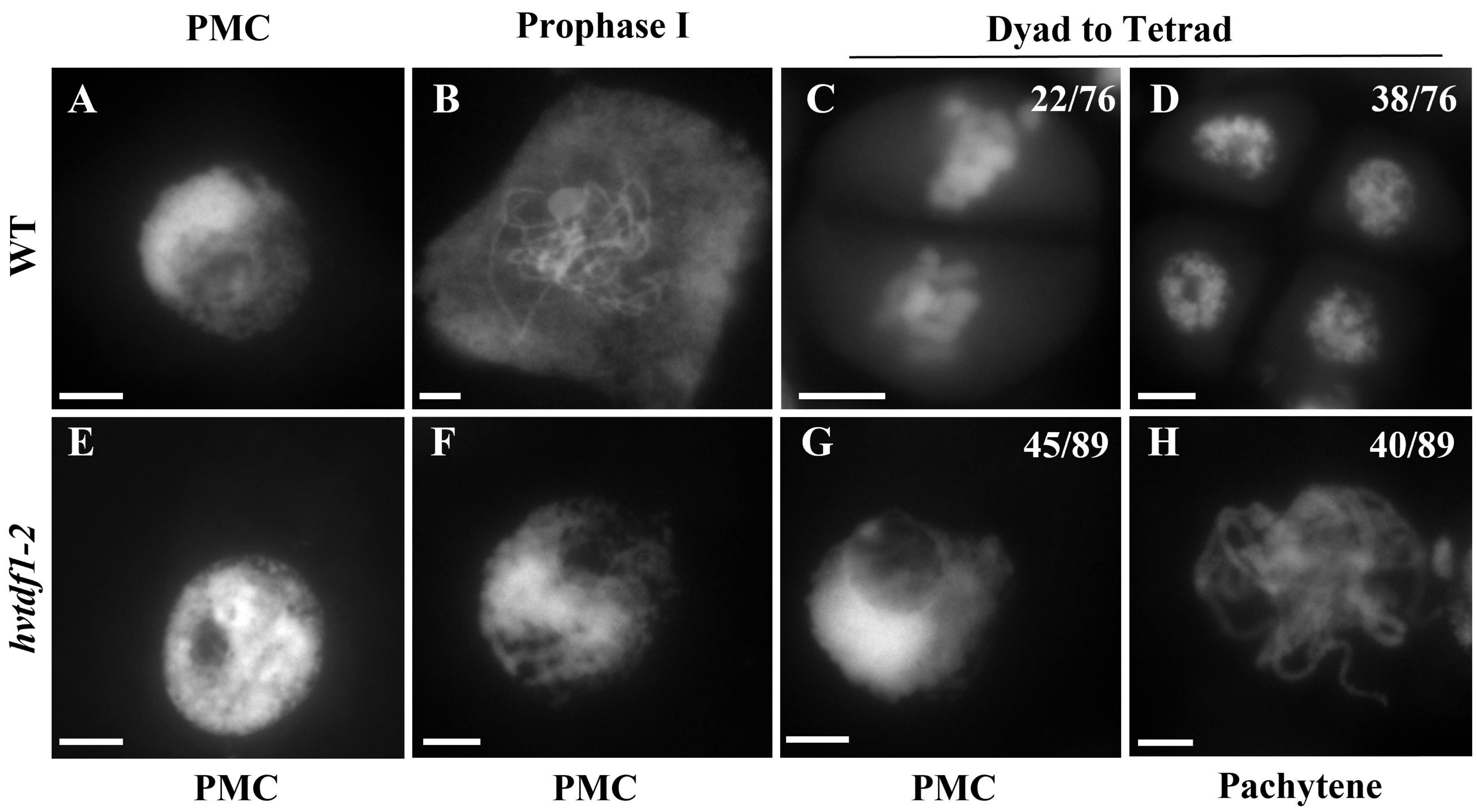

Fig. S3. The meiosis process in wild-type and *hvtdf1-2* male meiocytes from equivalent sized anthers at different development stages indicating different progression rates. (A, E) Pollen Mother Cells; (B, F) Prophase I; (C, G) Dyad; (D, H) Tetrad. Bar= 5µm. Numbers represent the counted cells from each stage.

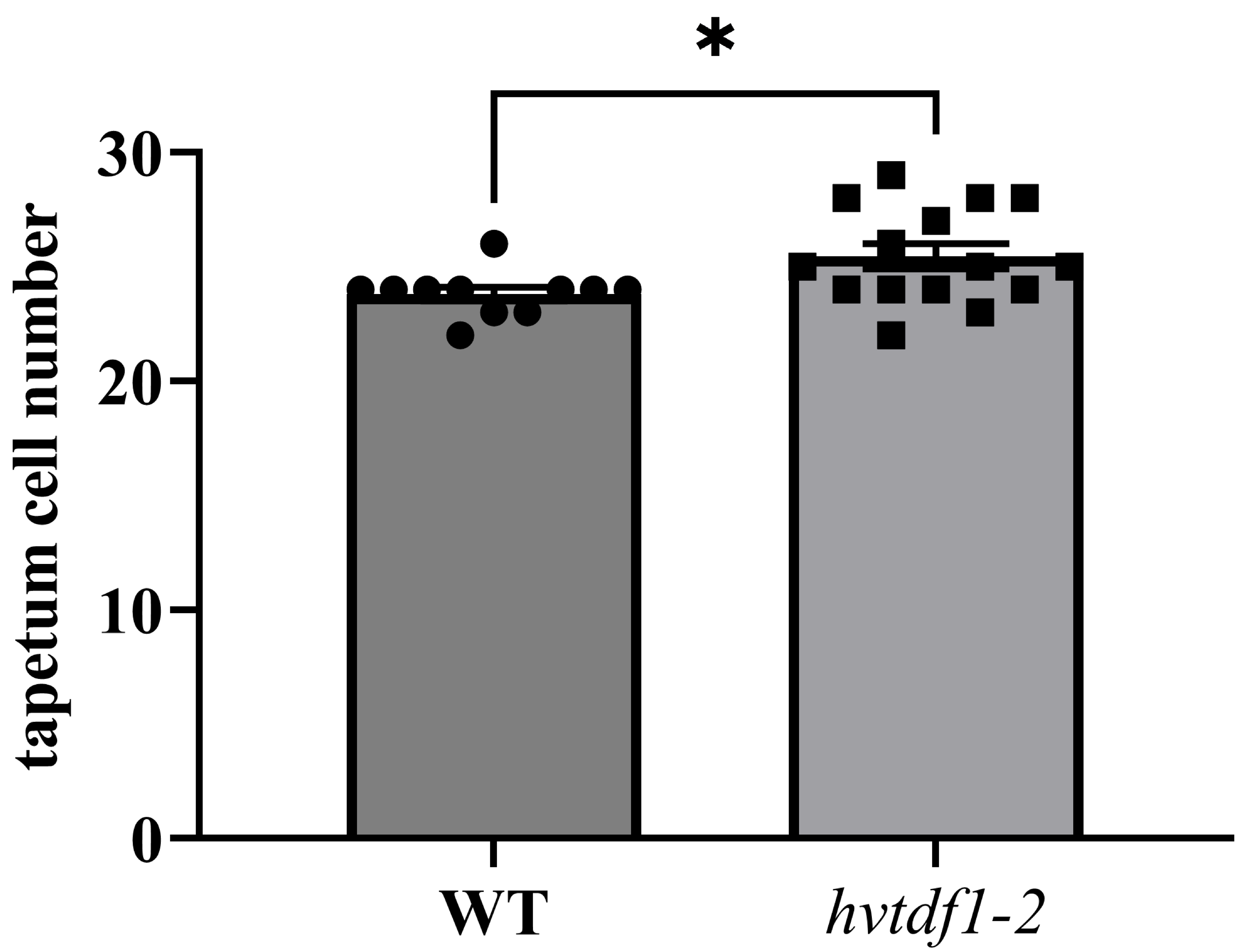

Fig. S4. Tapetal cell number between wild-type and *hvtdf1-2* based on cell counts from cross-section analysis at stage 8-9; only a slight increase (7%; *P<0.05) of tapetal cell number was observed in the mutant.

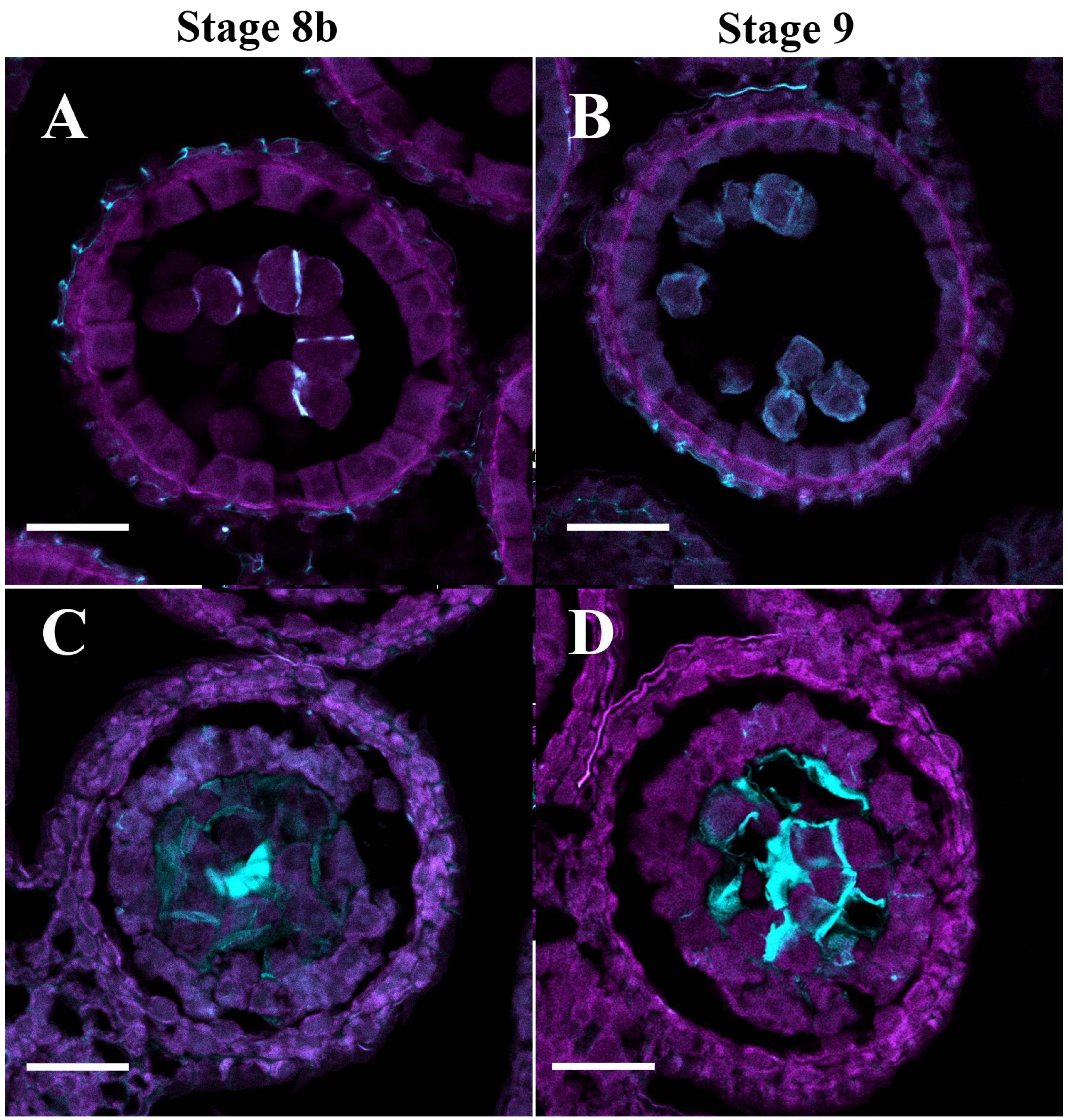

Fig. S5. Analysis of callose distribution in wild-type and *hvtdf1-2* anthers. (A-B) wild-type and (C-D) *hvtdf1-2* anther stained with aniline blue at stage 8b and stage 9, respectively. The cyan colour indicates the signal from the stained callose; the purple colour shows the autofluorescence of barley anthers. The wild-type tetrads and newly released single microspores are surrounded by callose, however the mutant line shows irregular callose accumulation in the middle of the anther. Bar = 30μm.

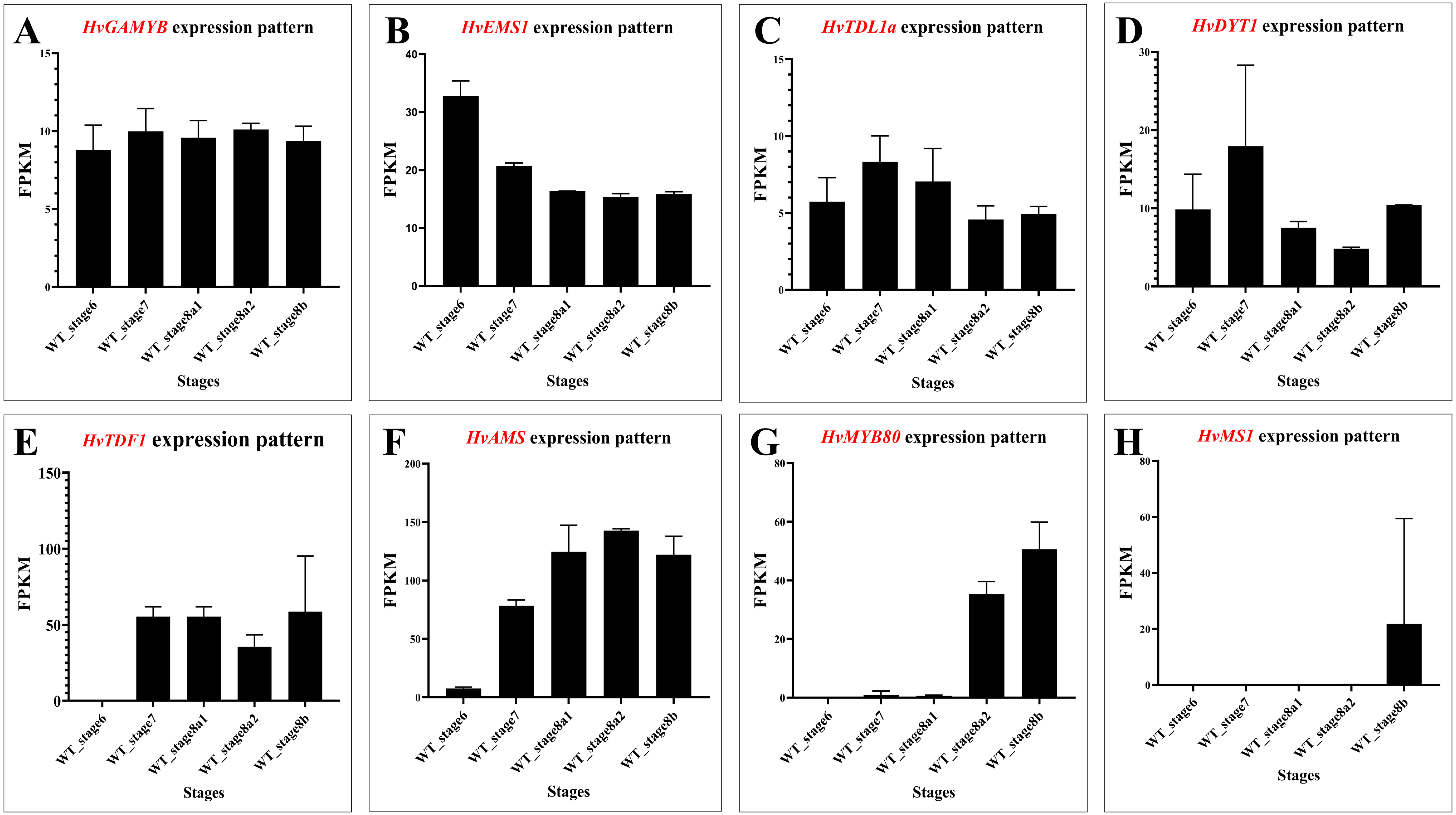

Fig. S6. The RNA-seq expression analysis of putative orthologous barley tapetum transcription factors in wild-type from stage 6 to stage 8b. (A) *HvGAMYB*, (B) *HvEMS1*, (C) *HvTDL1a*, (D) *HvDYT1*, (E) *HvTDF1*, (F) *HvAMS*, (G) *HvMYB80 and* (H) *HvMS1*. Error bar is the standard error of two biological replicates.

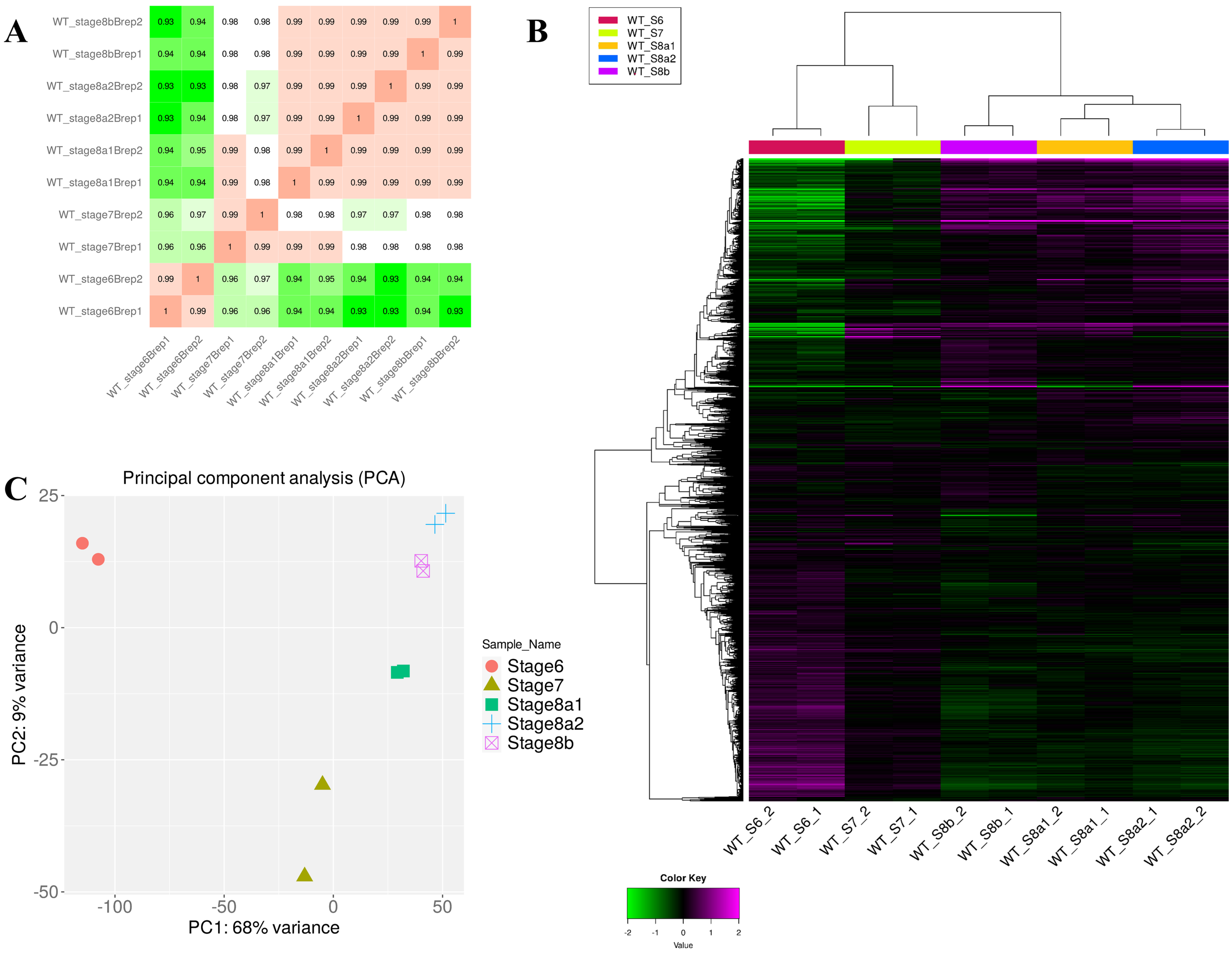

Fig. S7. The correlation and distance between different wild-type RNA-seq samples. (A) The top 75% genes correlation matrix, (B) the hierarchal cluster with heatmap among wild-type samples, (C) the PCA analysis of wild-type samples.

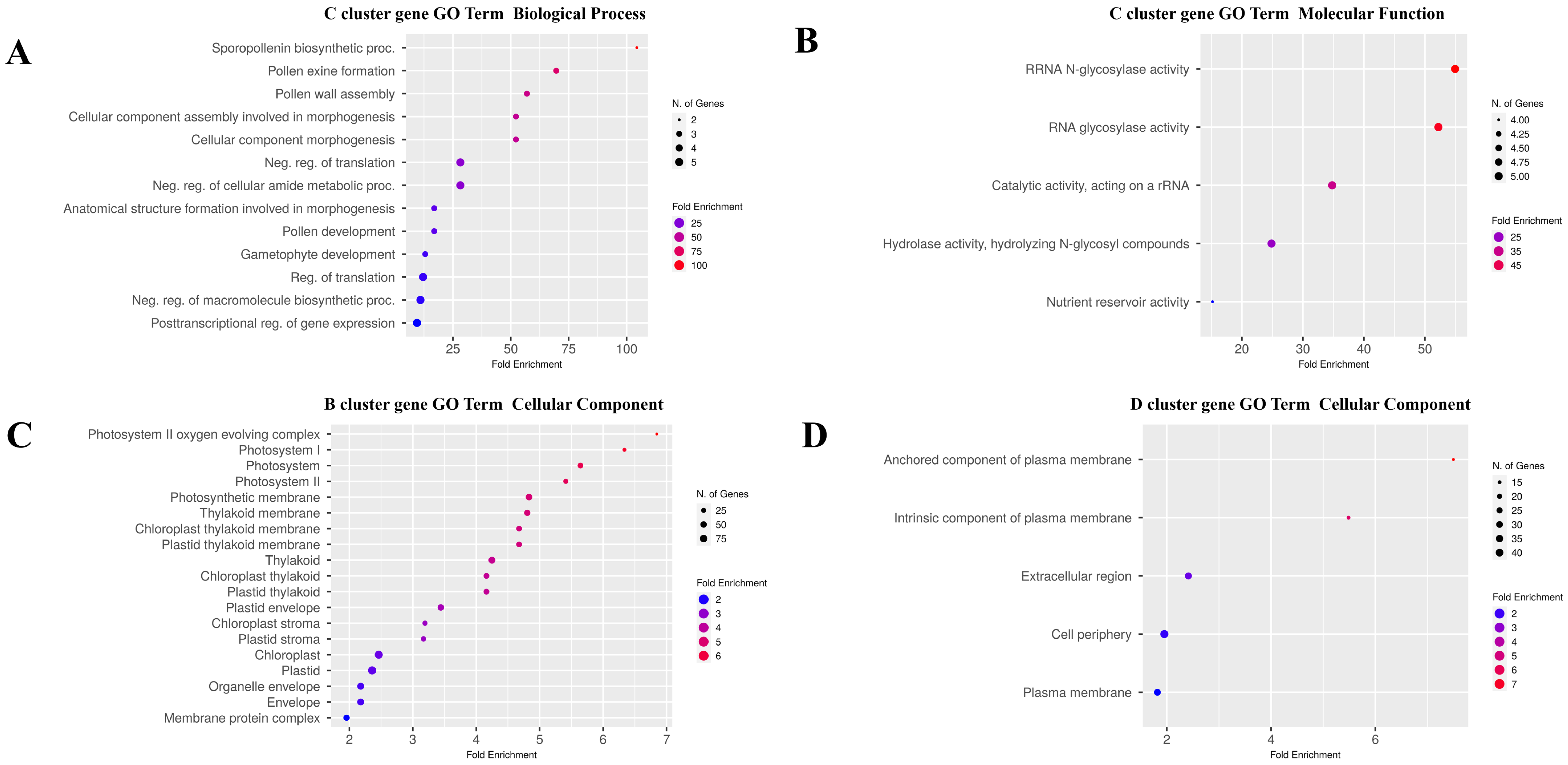

Fig. S8. GO Term analysis results of different gene sets from K-means clusters. (A) The C cluster biological process, (B) the C cluster molecular function, (C) the B cluster cellular component, and (D) the D cluster cellular component.

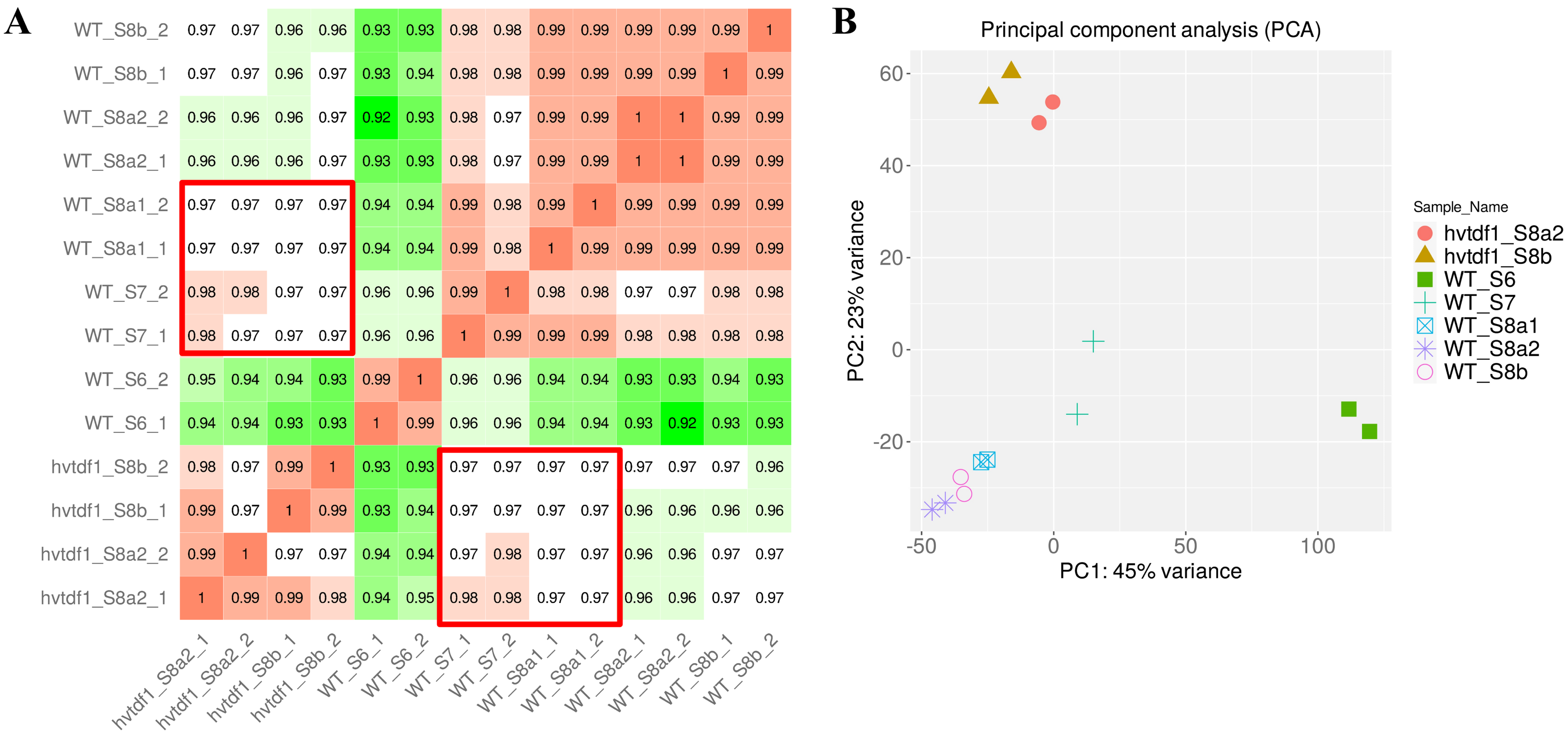

Fig. S9. PCA and correlation matrix analysis results between wild-type and *hvtdf1* mutant. (A) The top 75% genes correlation matrix, (B) PCA analysis.

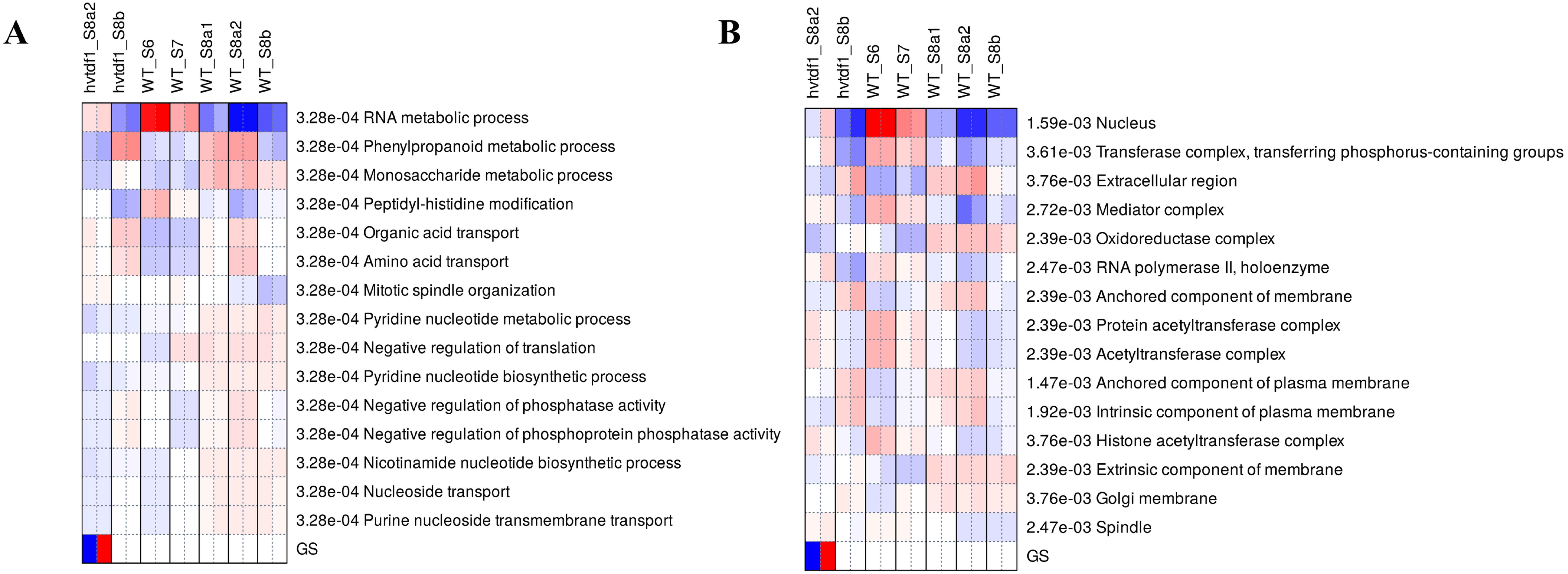

Fig. S10. The heatmap result of biological process and cellular component groups conducted with PGESA package. (Red and blue colour representing the activated and repressed pattern based on analysis data, respectively).

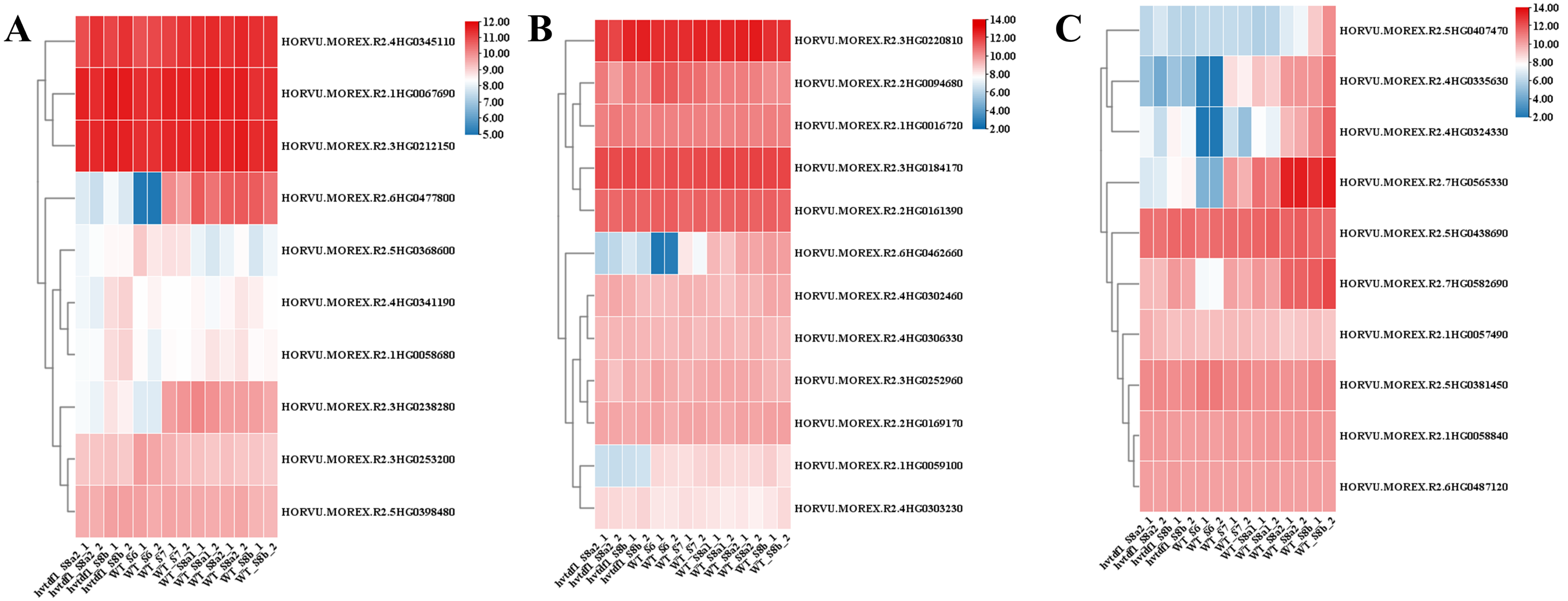

Fig. S11. The heatmap of gene expression pattern from PGSEA analysis results. (A) The expression pattern of genes involved in oxidoreductase acting on NAD(P)H pathway, (B) the expression pattern of genes involved in cation:cation antiporter activity, (C) the expression pattern of genes involved in pollen wall formation. Blue and red colour representing the down- and up-regulated, respectively.

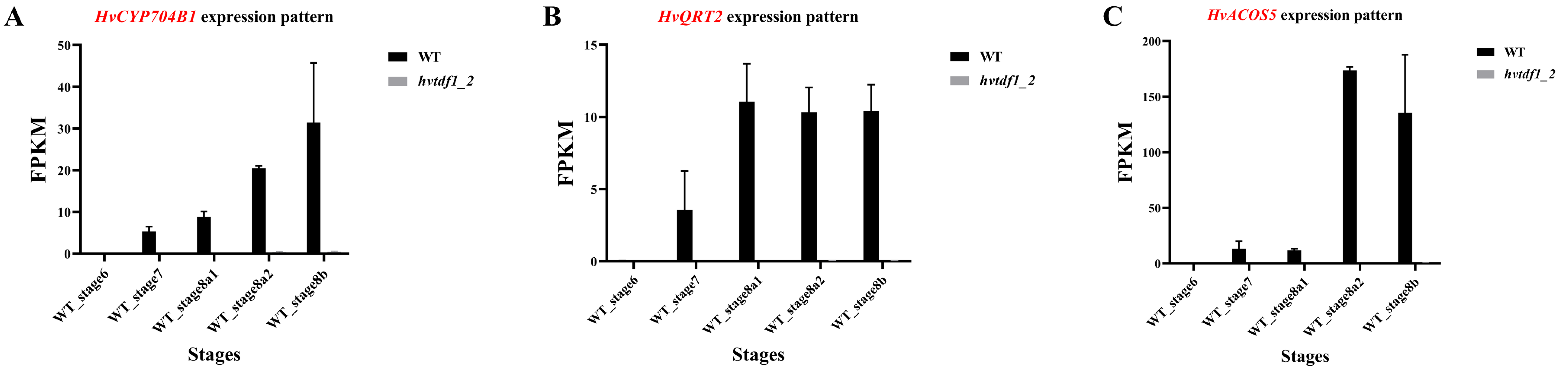

Fig. S12. The RNA-seq expression analysis of putative orthologous downstream genes of barley TDF1. (A) *HvCYP704B1*, (B) *HvQRT2*, (C) *HvACOS5*. Error bar is the standard error of two biological replicates.

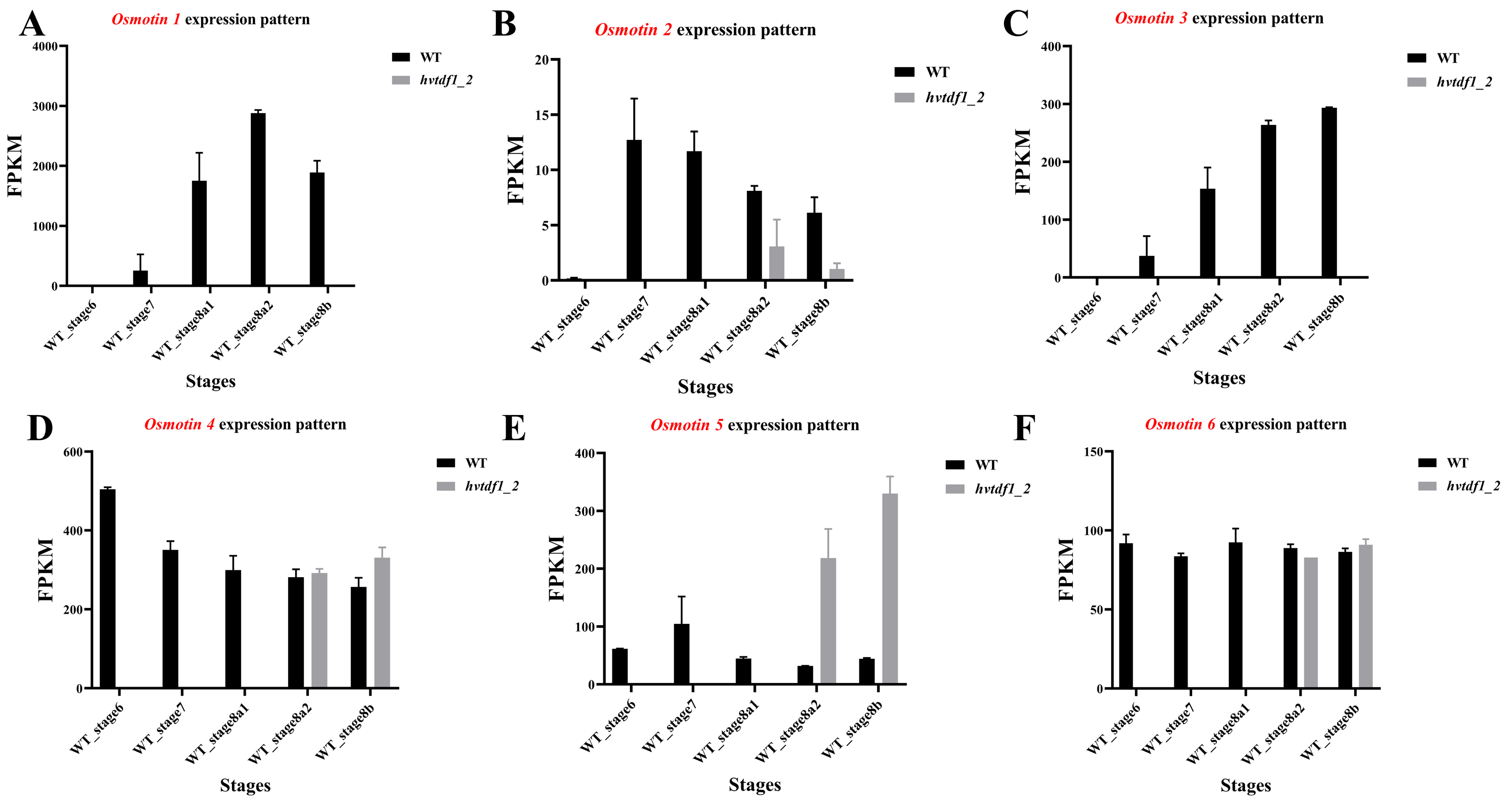

Fig. S13. The RNA-seq expression analysis of osmotin protein family genes in wild-type and *hvtdf1-2*. (A) *Osmotin 1*, (B) *Osmotin 2*, (C) *Osmotin 3*, (D) *Osmotin 4*, (E) *Osmotin 5*, (F) *Osmotin 6*. Error bar is the standard error of two biological replicates.

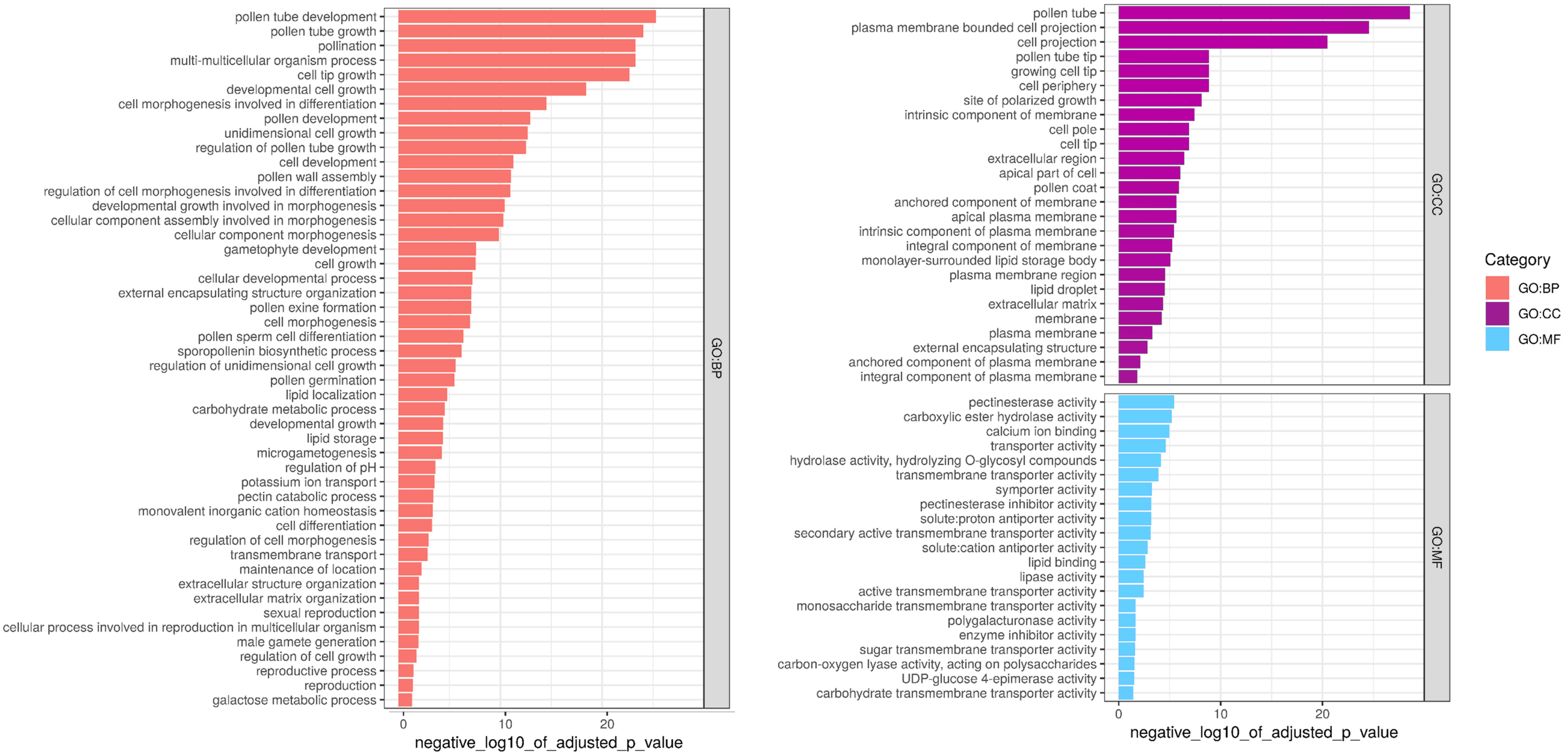

Fig. S14. GO Term analysis of the down-regulated genes from *attdf1* microarray data.

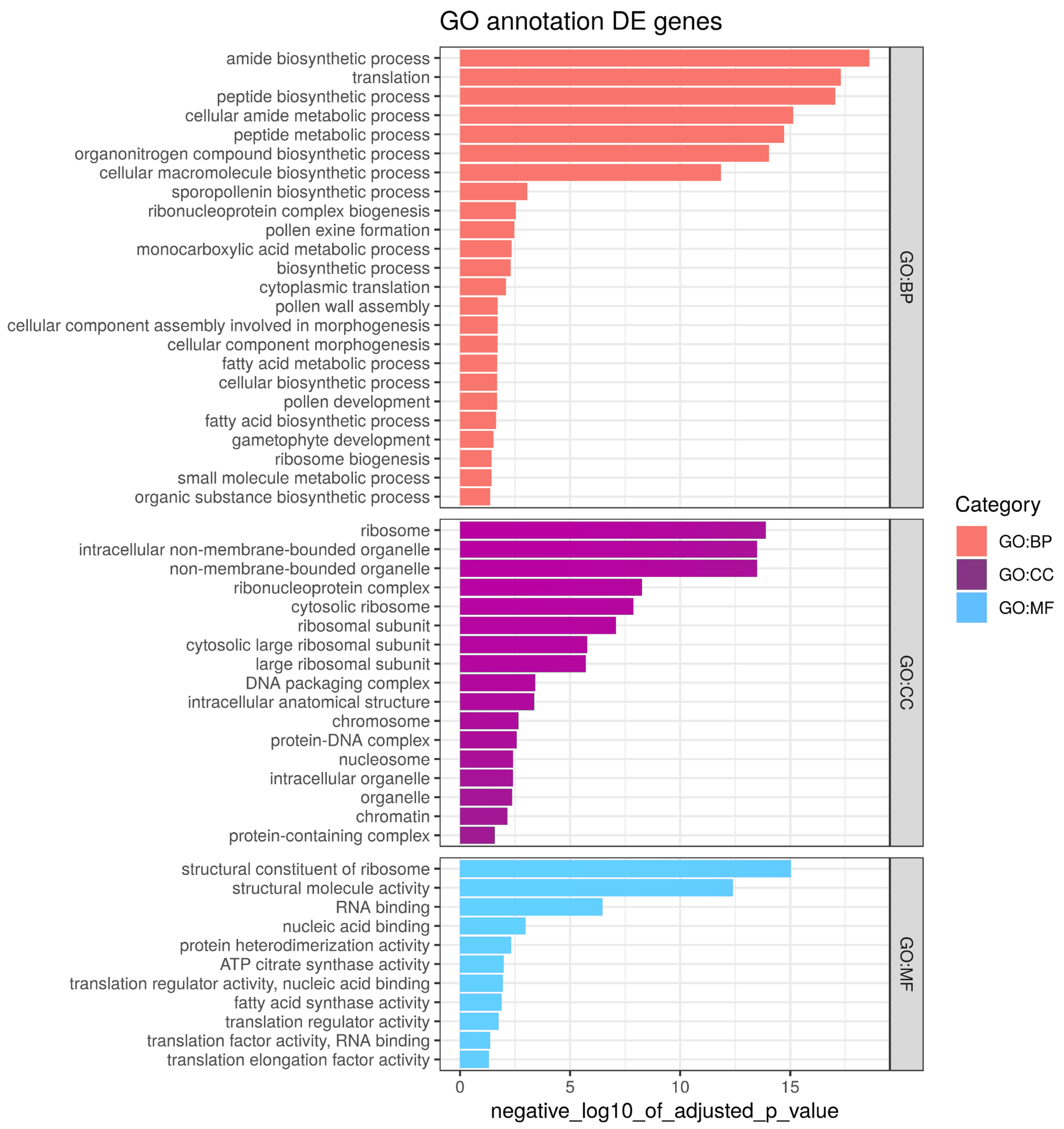

Fig. S15. GO Term analysis of the down-regulated genes from the comparison between *hvtdf1* and wild-type samples from stage8b. Gene Ontology (GO) Category- BP: Biological Process, CC: Cellular Component, MF: Molecular Function.

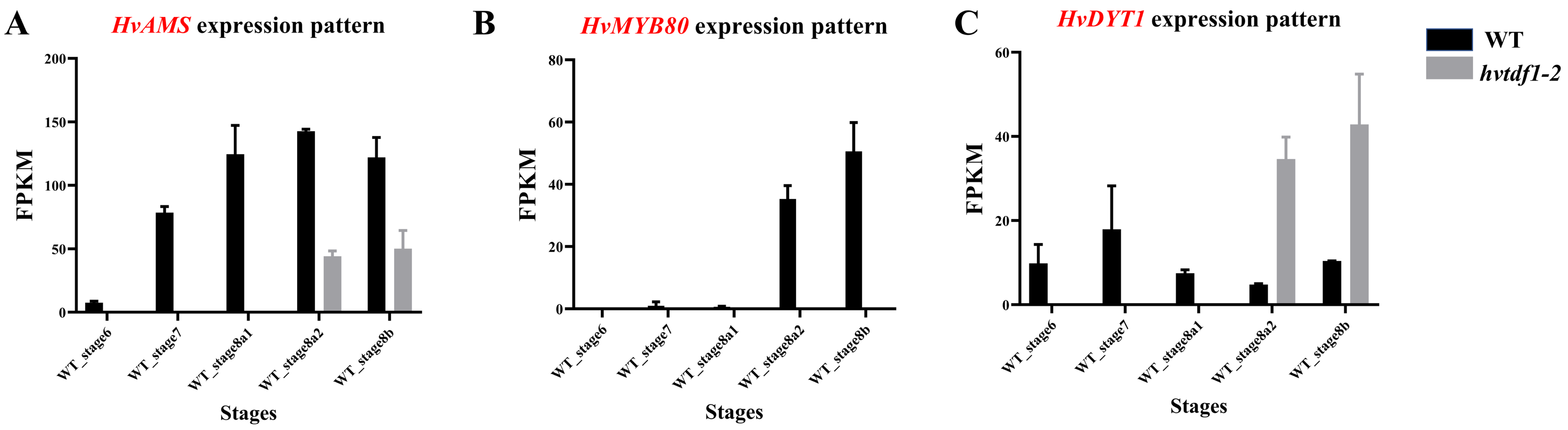

Fig. S16. The expression pattern of putative barley orthologous genes from wild-type (black) and *hvtdf1* mutant (grey). (A) *HvAMS*, (B) *HvMYB80*, (C) *HvDYT1*. Error bar is the standard error of two biological replicates.
